# Mechanical and gas adsorption properties of graphene and graphynes under biaxial strain

**DOI:** 10.1038/s41598-022-27069-y

**Published:** 2022-12-27

**Authors:** Raphael B. de Oliveira, Daiane Damasceno Borges, Leonardo D. Machado

**Affiliations:** 1 Departamento de Física Teórica e Experimental, Universidade Federal do Rio Grande do Norte, Natal, RN 59072-970 Brazil; 2 Physics Institute, Federal University of Uberlandia, Uberlandia, MG 38408-100 Brazil

**Keywords:** Atomistic models, Mechanical properties, Graphene, Two-dimensional materials

## Abstract

The exceptional properties of two-dimensional (2D) solids have motivated extensive research, which revealed the possibility of controlling many characteristics of these materials through strain. For instance, previous investigations demonstrated that compressive deformation could be used to direct the chemisorption of atomic hydrogen and oxygen. Still, to our knowledge, there is no work detailing how strain affects the adsorption isotherms of 2D materials and the adsorption properties of materials such as the graphynes, which are monolayers composed of sp and sp$^2$ carbon atoms. In the present work, we analyze how biaxial tensile deformation changes the adsorption properties of four 2D materials (graphene, $\alpha$-graphyne, $\beta$-graphyne, and $\gamma$-graphyne). To achieve this, we perform Monte Carlo Grand Canonical calculations to obtain the adsorption isotherms of H$_2$, CO$_2$, and CH$_4$ on the monolayers with and without strain. And, to apply the deformation, we carry out Molecular Dynamics simulations. We find a substantial reduction in the amount of gas adsorbed on the monolayers for nearly all gas–solid combinations. This is particularly true for graphene, where 14.5% strain reduces the quantity of H$_2$/CO$_2$/CH$_4$ by 44.7/64.1/41.7% at P $=$ 1 atm. To understand the results, we calculate adsorption enthalpies and analyze the gas distribution above the monolayers. We also characterize the mechanical properties of the considered solids under biaxial deformation. Finally, a comparison of pore sizes with the kinetic diameters of various gases suggests applications for the graphynes, with and without strain, in gas separation.

## Introduction

The isolation of graphene^1^ and the discovery of its exceptional properties—such as its very high electron mobility^2^, ultimate tensile strength^3^, and thermal conductivity^4^—has sparked the search for other two-dimensional (2D) materials. The graphynes, which are carbon monolayers composed of atoms with sp and sp$^2$ hybridization, are one 2D material family that has received attention since then. Baughman et al. initially predicted these solids in 1987^5^, but the first member of this family, the graphdiyne, was only synthesized in 2010^6^. Since then, the synthesis of $\gamma$-graphyne was also successful^7^, and other experimental works proposed using $\gamma$-graphyne in battery^8^ and supercapacitor applications^9^. Regarding the electronic properties of the graphynes, theoretical investigations revealed that some are semiconductors with moderate gaps^10^, while others feature Dirac cones in their electronic band structure^11^. And, in the case of $\gamma$-graphyne, calculations showed that strain could close the gap of this semiconductor and make Dirac cones appear^11^. Other simulations also characterized the mechanical properties of various graphynes, particularly under uniaxial strain.

We mentioned above that strain can modify the electronic properties of $\gamma$-graphyne, but deformation can also change properties in other materials. For instance, biaxial strain closes the bandgap of MoS$_2$ monolayers, turning this semiconducting solid metallic^12^. Deformation can also control the bandgap of other TMDs and phosphorene^13^. However, the magnitude of the bandgap change depends on the type of strain considered (uniaxial, biaxial, or vertical)^13^. Furthermore, the magnetic^14^ and thermal properties^15^ of certain 2D materials can be controlled through deformation.

Of relevance to the present work, strain in porous 2D materials can also modify the size and shape of their pores. For instance, by applying uniaxial deformation, it is possible to control diffusion rates in porous graphene, with potential applications in gas separation^16^. Similarly, biaxial strain increases the size of graphenylene pores, allowing the separation of different gas mixtures at particular pore sizes^17^. And by manipulating pore size using biaxial strain, it is possible to control water permeability in porous 2D materials while blocking the diffusion of salt ions dissolved in the water^18,19^. Finally, it is possible through deformation to change the adhesion in a covalent organic framework, and through this effect, it is possible to pick up and drop off other 2D materials^20^. This control of a material’s properties using strain has been termed strain engineering^13^, and here we investigate the effect of deformation on the adsorption properties of graphene and $\alpha$-, $\beta$-, and $\gamma$-graphyne. For each material, we consider their interaction with H$_2$, CO$_2$, and CH$_4$. We selected these gases for their energy applications (H$_2$ and CH$_4$) and their contribution to global warming (CH$_4$ and CO$_2$).

The adsorption of the gas molecules considered here on graphene has been examined experimentally and theoretically. Experimental investigations on the adsorption of H$_2$ on graphene revealed an uptake of up 3 wt%, but only at low temperatures (77 K and 1 atm) or high pressures (298 K and 100 atm)^21^. At the same time, theoretical studies have determined the most stable configuration for the adsorbed H$_2$^22^, determined its binding energy curve^23^, and the adsorption isotherms^24^. Regarding the adsorption of CO$_2$ on graphene, experiments by Ghosh et al. obtained high gas uptake at 195 K and 1 atm^21^, while combined experimental and theoretical investigations examined the adsorption of CO$_2$ on graphene at lower temperatures (30 K) and obtained its desorption energy^25^. Concerning methane, its adsorption on graphene was investigated experimentally at temperatures ranging from 253.15 to 293.15 K and pressures ranging from 0 to 8 MPa^26^. The authors found similar adsorption characteristics for CH$_4$ on graphene and activated carbon. Meanwhile, density functional theory (DFT) calculations studied the most stable configuration, the adsorption energy, and the charge transfer for methane on graphene^27^. Finally, note that various studies have also considered doping or decorating graphene with other elements or molecules to (1) improve its H$_2$ storage properties^28,29^ and (2) CO$_2$ or CH$_4$ adsorption energies, to improve its performance in sensors^27,30,31^.

On the other hand, the adsorption of gases on graphyne sheets has received less attention, and to our knowledge, there is no experimental work on this topic. However, theoretical studies have obtained the adsorption energies, preferential adsorption sites, and the change transfer for H$_2$ and CO$_2$ on $\gamma$-graphyne^32^. Graphtriyne sheets have also been considered, and the results indicate that single layers of this solid could be used to separate CO$_2$ and N$_2$, while triple layers present high uptake of CO$_2$^33^. Meanwhile, investigations on graphynes decorated with other elements are more common, aiming to improve: (1) hydrogen storage^34–38^, (2) methane storage^39^, and (3) CO$_2$ capture capacity^40–43^.

Some studies have already considered applying strain to graphene layers to alter their adsorption properties, but these have focused on atomic hydrogen or oxygen chemisorption. Initial theoretical proposals introduced out-of-plane deformation in graphene to control the hydrogen-graphene binding energy. The authors discovered that convex regions are favorable for atomic hydrogen adsorption^44^. Next, a DFT study proposed applying compressive strain to create ripples in graphene, creating convex regions where hydrogen preferentially adsorbs, allowing for the control of the bandgap^45^. Experimental studies then confirmed the preferential adsorption of atomic hydrogen on convexly curved areas in graphene grown on SiC and showed that this uneven adsorption does not occur in graphene bilayers^46,47^. Following simulation studies investigated the structure and electronic properties of pristine and hydrogenated corrugated graphene, intended to mimic graphene on SiC^48^. Regarding oxygen chemisorption, the simulation proposal was to use strain to introduce ripples, leading to preferential O adsorption on regions with high local curvature, followed by oxidative cutting of the resulting structures to produce graphene nanoribbons^49^. The effect of strain on adsorption properties has also been considered for transition metal dichalcogenides (TMDs) monolayers. Theoretical studies have introduced biaxial deformation to control the interaction of different TMDs with hydrogen^50^, NO$_2$^51,52^, and CO^53^. Sensor applications have been proposed for this effect^51,52^.

However, to our knowledge, there is presently no work detailing how strain affects the adsorption isotherms of 2D materials and no study examining how deformation modifies the adsorption properties of the graphynes. Here, we investigate the adsorption properties of three gases on relaxed and strained monolayers of graphene and graphyne. For each combination of solid and gas, we calculate adsorption isotherms and enthalpies of adsorption. We also examined the structural changes and obtained the mechanical properties of all considered monolayers under biaxial strain.

## Results and discussion

**Figure 1:**
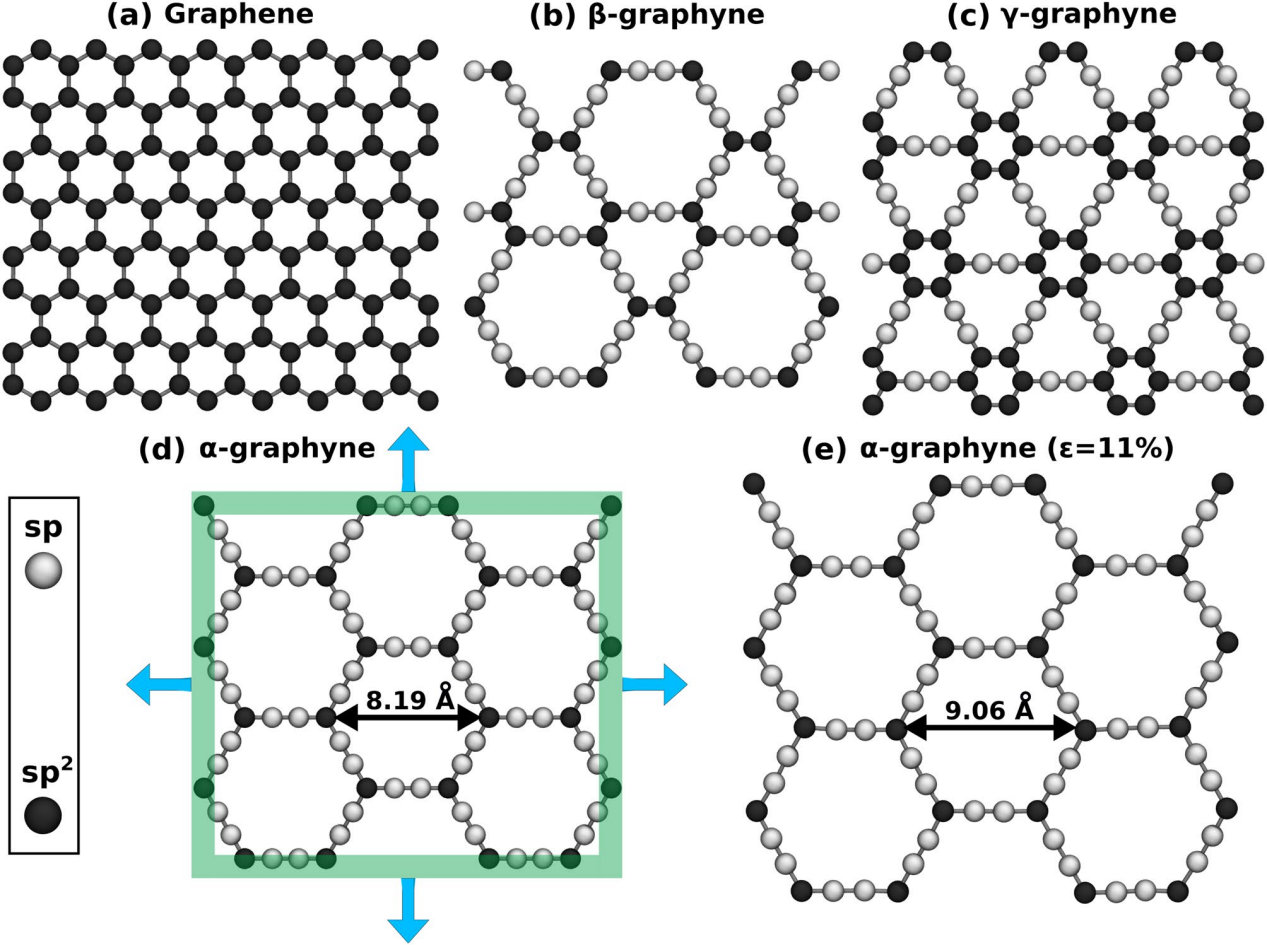
(**a**–**d**) Investigated structures. We deform both planar directions equally in this work, as indicated in (**d**). (**e**) presents the $\alpha$-graphyne monolayer after the application of 11% biaxial strain. Sp/sp$^2$ atoms are represented using white/black spheres in this figure.

The methodology used to determine the effect of strain on the adsorption properties of $\alpha$-, $\beta$-, $\gamma$-graphyne, and graphene had two stages. First, we used Molecular Dynamics simulations (MD) to apply biaxial strain to the investigated materials; then, we carried out Grand Canonical Monte Carlo (GCMC) simulations to calculate adsorption isotherms for the structures with and without deformation. Details are presented in the Methods section. Figure 1 illustrates the simulated 2D structures.

**Figure 2:**
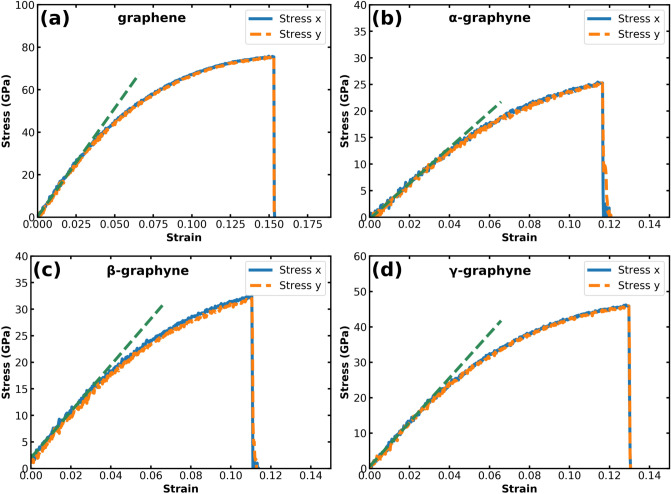
Stress–strain curves for graphene and the three graphynes considered at T = 298 K. We present stress values along both the *x* and the *y* directions in this figure. However, notice that stress values are equal for both directions, in accordance with other reports that investigated 2D materials under biaxial strain^56,57^.

Let us begin the discussion of the results by examining the mechanical properties of graphene and the graphynes. Figure 2 shows the stress–strain curve of each material when applying equal biaxial deformation at ambient temperature (T $=$ 298 K). Notice that the curves are very similar for both in-plane directions. Thus, we can obtain the biaxial modulus by calculating the slope of the elastic region (i.e. the linear region) in one direction, following a similar procedure reported in the literature^54–57^. Table 1 shows our main results: the biaxial modulus, ultimate strength, and ultimate strain. The first quantity relates to the materials’ stiffness, while the second and third correspond to the maximum stress and strain the material can withstand, respectively. Comparing the results for the different materials, we find the same trends observed for the mechanical properties under uniaxial strain reported by Pei et al. in the case of graphene^58^ and Zhang et al. in the case of $\alpha$-, $\beta$-, and $\gamma$-graphyne^59^. Overall, graphene is the stiffest and strongest material, with a biaxial modulus of 1020 GPa and enduring stress values of up to 75.8 GPa. On the other hand, the $\alpha$-graphyne is the weakest and least stiff solid. Finally, all materials withstand strain above 10% before fracture, even under biaxial deformation.

To avoid thermal perturbation on the stress–strain curve and to compare with values reported in the literature, we have also applied equal biaxial deformation at cryogenic temperatures (see Supplementary Information, Fig. S1 and Table S1). At T $=$ 10 K, the biaxial modulus and strength are slightly higher for all solids, whereas the ultimate strain is considerably higher (by at least 15%). Table 1 also provides results from previous works that investigated graphene and $\gamma$-graphyne under biaxial strain. We find the values obtained here are lower than those previously reported. However, the literature values correspond to the mechanical properties at T = 0 K^55,57,60^. Two of those studies considered the variation of these properties with the temperature. They found that the biaxial modulus^55^, the ultimate strain, and the ultimate strength of graphene decrease as the temperature increases^57^, in agreement with our findings.

**Table 1:** Mechanical properties of graphene and the graphynes under biaxial strain.

	Biaxial modulus (GPa)	Ultimate strain	Ultimate strength (GPa)
Graphene^55,57^	1185$^*$	0.204	97.0
Graphene (this work)	1020	0.153	75.8
$\alpha$-graphyne (this work)	335.6	0.117	25.4
$\beta$-graphyne (this work)	436.5	0.111	32.5
$\gamma$-graphyne^60^	–	0.18	60.7$^*$
$\gamma$-graphyne (this work)	626.6	0.130	46.2

Results marked with $^*$ were converted from N/m to GPa assuming a thickness of 3.4 Å for the monolayers. In this table, we show our results for T = 298 K, while the results from the literature are for T = 0 K.

Before investigating the effect of the material deformation on the adsorption properties of gases, let us discuss how the pore geometry changes with deformation. In the current and the following discussions, we refer to unstretched/stretched structures with the label *without*/*with* strain. Note that we assumed different deformations for each structure because the ultimate strain varies between monolayers, and the value used for each solid is given in Table 2. Figure 1d,e illustrate the example of $\alpha$-graphyne without and with strain. Notice that when biaxial strain is applied, the pore size must increase, and the hexagonal pore maintains its shape. Thus, we calculated the pore diameter using the definition implemented in the Zeo++ software package^61^, which corresponds to the diameter of the largest possible sphere included in the pore. In the methodology used in this code, the atomic radius is considered, avoiding repulsive interactions^61^. The effect of the strain on the pore diameter is evident and can be observed in Table 2. This geometrical property is relevant to the selectivity of these membranes and can be directly compared with the kinetic diameter of the molecule during a process called molecular sieving.

Inspecting the obtained pore diameters, we find that the triangular pores of $\gamma$-graphyne are too narrow to allow gases to pass through, even after deformation. For comparison, the kinetic diameter of H$_2$ is 2.89 Å and of He is 2.6 Å^62^. Indeed, the energy barrier to crossing a strained triangular hole does not vanish for the gases investigated here (see Fig. S20). On the other hand, the hexagonal pores of the $\alpha$- and $\beta$-graphyne are larger, allowing for the passage of some gases while blocking others. We analyze the case of $\alpha$-graphyne here since, in comparison to $\beta$-graphyne, the variation of its pore diameter is more significant. Prior to the application of strain, the pore diameter of $\alpha$-graphyne is close to the kinetic diameter of various gases, for instance: CO$_2$ (3.3 Å), O$_2$ (3.46 Å), H$_2$S (3.6 Å), N$_2$ (3.64 Å), CO (3.76 Å), and CH$_4$ (3.8 Å)^62^. Of these gases, CO and CH$_4$ have a kinetic diameter larger than the pore, and it might be possible to separate them from the other gases using $\alpha$-graphyne membranes. After the introduction of 11% strain, the pore diameter increases from 3.69 to 4.64 Å and becomes larger than the kinetic diameter of molecules such as ethylene (3.9 Å) and propane (4.3 Å)^62^. Since the pore diameter (*d*) varies continuously with the deformation, intermediate values might be useful to separate hydrocarbon gases. For instance, at $d=3.85$ Å, the pore is larger than the kinetic diameter of methane but smaller than the kinetic diameter of ethylene and propane.

One final note regarding the passage of gases through the graphyne membranes is that we compared energy profiles obtained using classical potentials with profiles obtained using Density Functional Methods in the Supplementary Information (Figs. S23, S24). While the DFT calculations support the assertions made above, they also reveal limitations of the classical methodology, such as underestimating the attractive interaction between the monolayers and the gases.

**Table 2:** Pore diameters and mass surface densities for the investigated monolayers before and after the introduction of strain.

	Graphene	$\alpha$-graphyne	$\beta$-graphyne	$\gamma$-graphyne
Strain	14.5%	11%	10%	12%
Pore diameter (Å)	–	3.69/4.64	3.97/4.54	0.85/1.47
Surface density (mg/m$^2$)	0.763/0.602	0.384/0.301	0.441/0.372	0.591/0.465

The strain value indicated in the first row corresponds to the deformation used in the GCMC simulations. The first/second number on each column corresponds to the structure without/with strain.We did not provide a pore diameter for graphene because it is a non-porous material.

**Figure 3:**
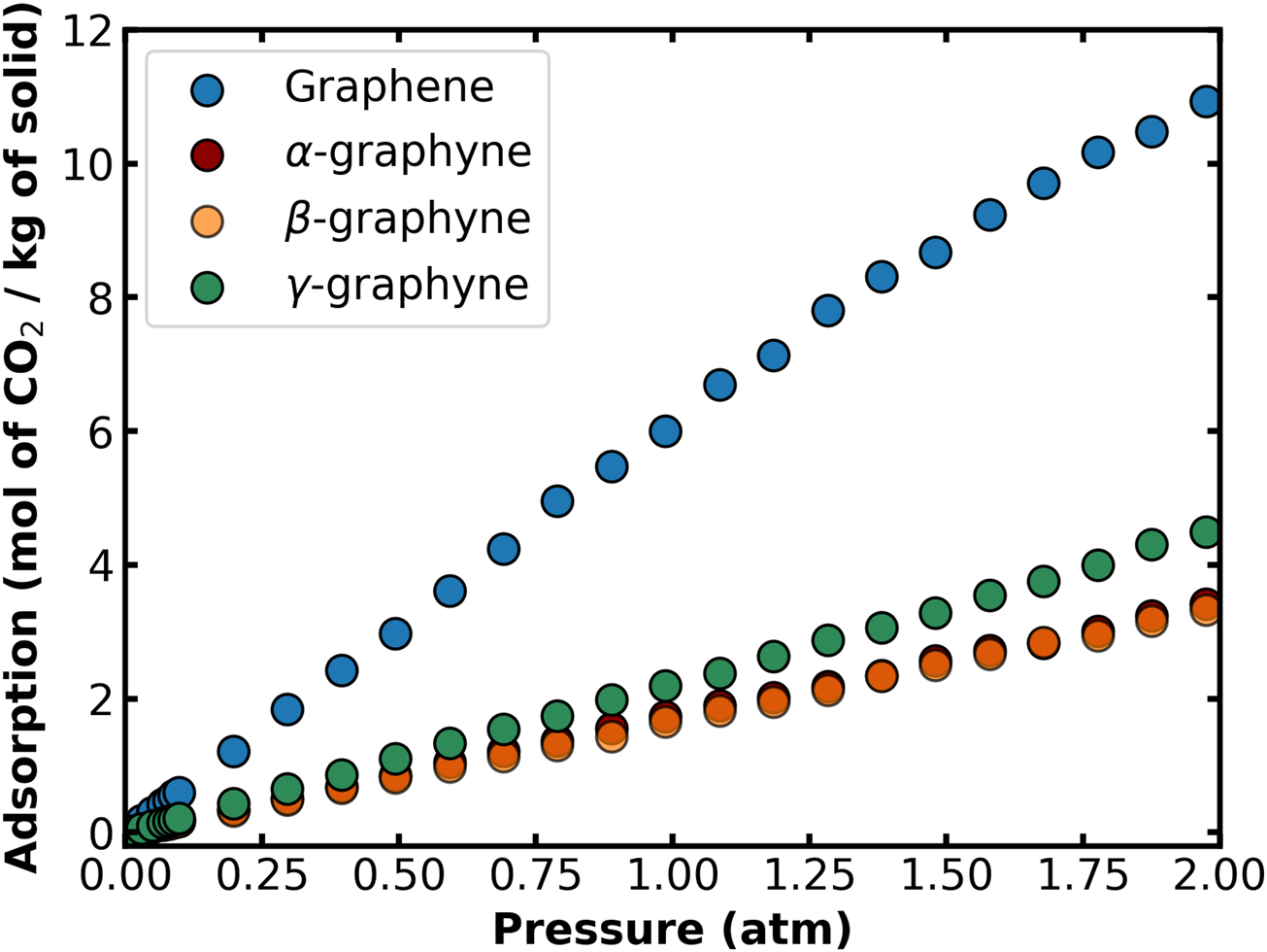
Adsorption isotherms of CO$_2$ on graphene and the three graphynes without strain (T $=$ 298 K). The isotherms of $\alpha$- and $\beta$-graphyne overlap, and it is hard to differentiate them.

The material deformation may also affect their affinities with gases. To investigate that, we performed grand-canonical Monte Carlo simulations to predict the adsorption isotherms of CO$_2$, CH$_4$, and H$_2$ on the rigid solids with and without strain. Figure 3 shows the CO$_2$ adsorption isotherms on the relaxed graphene and graphynes at T $=$ 298 K. These results show weak gas-monolayer affinities with a low amount of gas adsorbed at low pressure. In general, the LJ parameters from UFF, as well as the gas models adopted in this work, appear to underestimate the guest-host interaction energy in comparison with other models, such as ILJ^63^ and CCSD^64^. From Fig. 3, we observe that the type of carbon material can significantly affect the CO$_2$ isotherm. For instance, the CO$_2$ loading on graphene at pressure P $=$ 1 atm is more than 2.7 times larger than that on $\gamma$-graphyne, which in turn holds an amount of gas $\approx$ 30% greater than either $\alpha$- or $\beta$-graphyne. An analogous analysis performed on the adsorption of H$_2$ and CH$_4$ revealed that graphene also holds the highest amount of H$_2$ and CH$_4$, whereas the three graphynes contain similar amounts of either gas. More details can be found in the Supplementary Information (Figs. S2, S3).

Furthermore, we compute the adsorption enthalpy at zero-loading, which is a measure of the heat released during the molecule adsorption and therefore provides a guide to the energy interaction between guest and host. Comparing the enthalpy values in Table 3 and the isotherms in Fig. 3, we observe that the amount of CO$_2$ adsorbed is directly related to the heat of adsorption. Since we are considering only homogeneous surfaces composed of the same atom of C, there should be a direct relationship between adsorption enthalpy and the number of interacting carbon atoms. For instance, the mass surface density of graphene is significantly larger than the densities of the graphynes (see Table 2), which justifies the higher adsorption enthalpy of this material. Except in the case of $\alpha$- and $\beta$-graphyne, the first material offers slightly higher adsorption enthalpy while the second one offers higher density. Indeed, contrary to the other materials, the $\alpha$-graphyne has the first adsorption site centered on the hexagonal pore in almost the same plane as the monolayer (say, at a height below 2 Å from the surface). This statement is supported by the 2D map of CO$_2$ distribution on the vicinities of $\alpha$-graphyne that clearly shows the presence of CO$_2$ in the center of the pore (see Fig. S10). A similar effect does occur in the large pores of $\beta$-graphyne, but it is less pronounced since there are fewer hexagonal pores on this structure.

**Table 3:** Adsorption enthalpies at T $=$ 298 K for CO$_2$, CH$_4$ and at T $=$ 77 K for H$_2$ on graphene and graphynes without and with strain.

Enthalpy (kJ/mol)			
	CO$_2$	CH$_4$	H$_2$
**Graphene**			
Without strain	− 18.78	− 13.49	− 5.17
With strain	− 13.35	− 9.30	− 3.85
$\alpha$-graphyne	CO$_2$	CH$_4$	H$_2$
Without strain	− 11.82	− 7.24	− 4.77
With strain	− 8.42	− 6.09	− 2.89
$\beta$**-graphyne**			
Without strain	− 10.69	− 6.68	− 4.05
With strain	− 8.94	− 6.46	− 3.00
$\gamma$**-graphyne**			
Without strain	− 13.45	− 9.46	− 4.01
With strain	− 9.54	− 6.84	− 3.01

**Figure 4:**
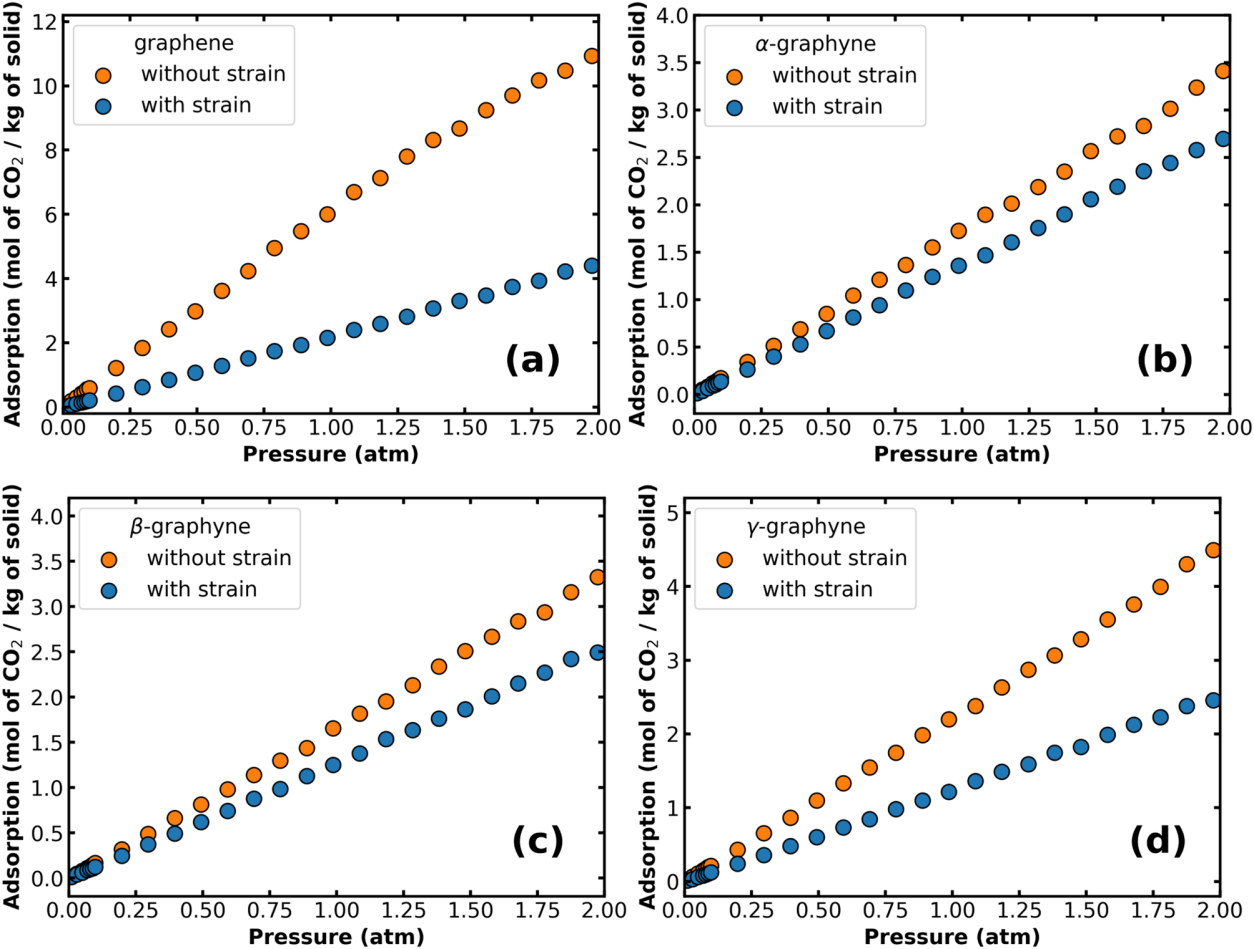
(**a**–**d**) Adsorption isotherms of CO$_2$ on graphene and the graphynes, comparing results with and without strain (T $=$ 298 K).

Now, let us consider the effect of the material’s deformation on the adsorption of CO$_2$. Figure 4 displays the isotherms for solids without and with strain. In all cases, we can observe a reduction in the isotherm when applying the strain. For instance, at P $=$ 1 atm, we find that strain reduces the CO$_2$ loading by 64.1%, 21.3%, 24.5%, and 44.7% for graphene, $\alpha$-, $\beta$-, and $\gamma$-graphyne, respectively. Also, for all materials, the enthalpy of adsorption decreased with the strain, as shown in Table 3. Similar trends were observed in the case of CH$_4$ and H$_2$ adsorption, and their isotherms can be found in the Supplementary Information (Figs. S4, S5). To illustrate general tendencies, we provide below the percent reduction in the adsorption of H$_2$ and CH$_4$ due to deformation at P $= 1$ atm:

- For H$_2$: 44.7% in graphene, 40.8% in $\alpha$-graphyne, 35.2% in $\beta$-graphyne, and 42.6% in $\gamma$-graphyne.
- For CH$_4$: 41.7% in graphene, 21.1% in $\beta$-graphyne, and 25.2% in $\gamma$-graphyne. In the case of $\alpha$-graphyne, the adsorbed amount increased by 3.9%.

Except for CH$_4$ in $\alpha$-graphyne, the deformation substantially reduced the quantity of adsorbed molecules on the surface of the monolayers. The main reason for that is related to the decrease in interaction site density (mass density), which directly affects the adsorption enthalpy as shown in Table 3. Furthermore, graphene is the material where the reduction is most accentuated, while in $\alpha$- and $\beta$-graphyne the reduction is less important. This last observation can be explained by the gain in the number of adsorption sites near the hexagonal pores after applying strain, as discussed below. Finally, the reduced adsorption enthalpy implies that less heat is required to remove the adsorbed gas from the monolayer, suggesting strain application as a mechanism for facilitating gas desorption.

**Figure 5:**
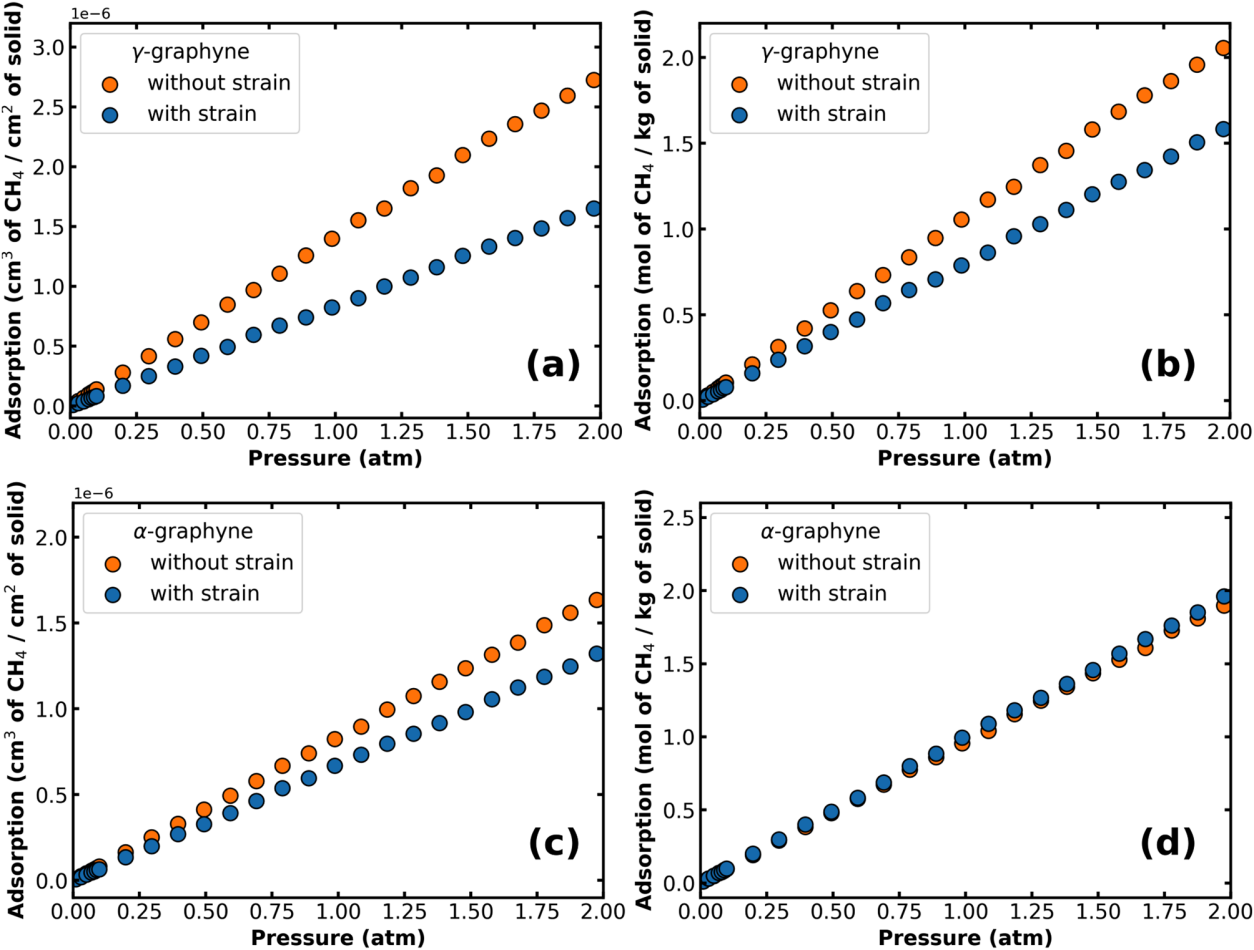
Adsorption isotherms of CH$_4$ on (**a**,**b**) $\gamma$-graphyne and (**c**,**d**) $\alpha$-graphyne, comparing results with and without strain and considering different adsorption units (T $=$ 298 K).

We have presented all isotherms up to now in units of mol of gas per kg of material, and there is a methodology issue behind this choice. Consider Fig. 5, which shows adsorption isotherms of CH$_4$ on $\gamma$-graphyne and $\alpha$-graphyne. Comparing Figs. 5a and 5b, we find that the percentage of reduction depends on which unit is adopted. For instance, at P $=$ 1 atm, the strain reduces CH$_4$ adsorption in $\gamma$-graphyne by 41.1% in units of cm$^3$ of gas per cm$^2$ of solid, while this reduction is only 25.2% in units of mol of gas per kg of solid. This unit dependence occurs because strain increases the surface area of the material, increasing the denominator in the unit where it is used. We opted to avoid using units of volume of gas per area of solid because this increase in the denominator is not related to an actual reduction in the amount of gas adsorbed on the material. Figure 5c,d highlight this point. Examining Fig. 5d, we find that the number of mols of CH$_4$ on $\alpha$-graphyne increased slightly with the deformation (we explain this result below). However, since the solid area increase is more important, we find an apparent reduction in the amount of gas adsorbed on the surface when using units of volume of gas per area of the monolayer. The same issue occurs if we consider the volume of the 2D material instead of its area.

**Figure 6:**
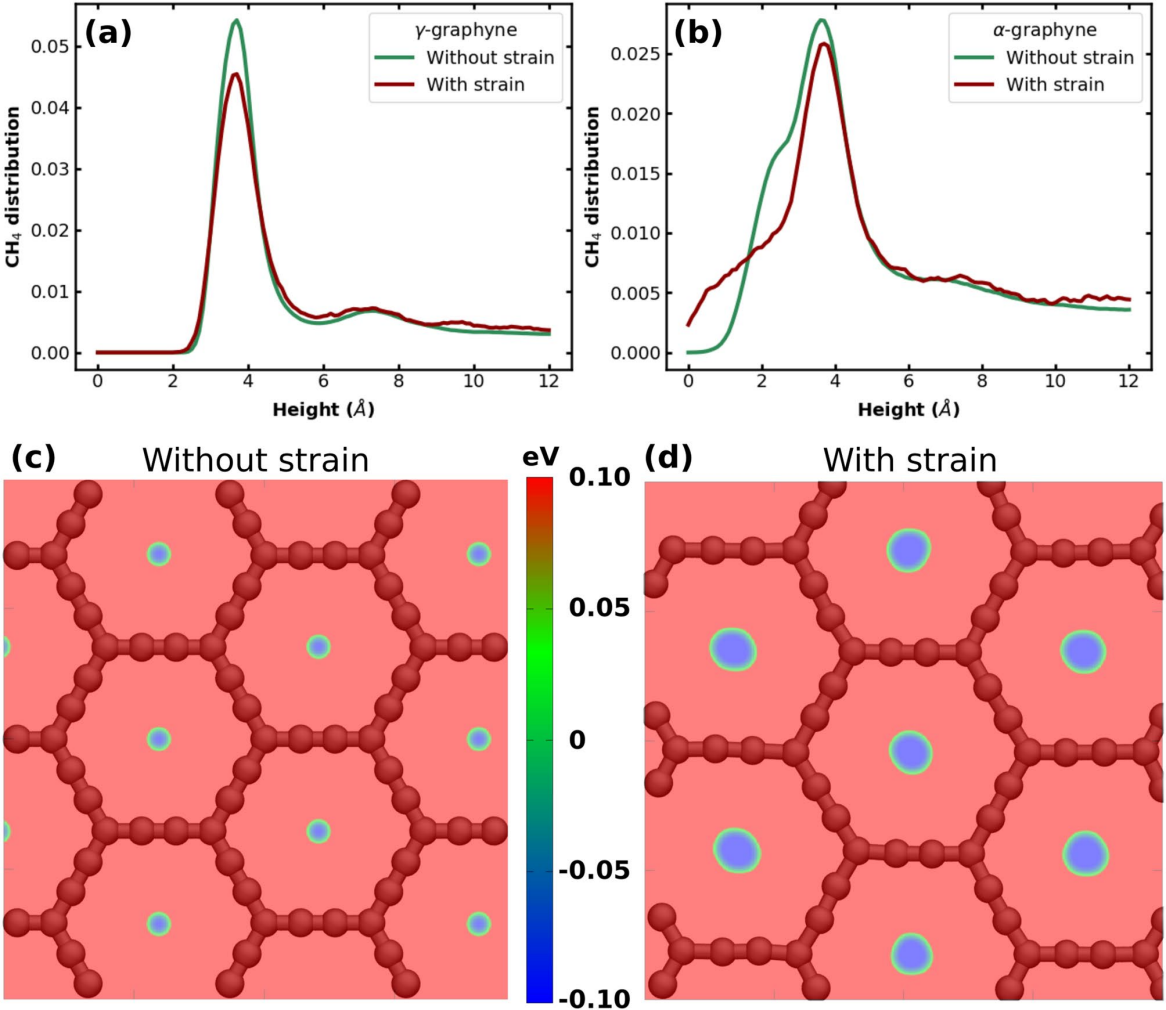
Histogram detailing the distribution of CH$_4$ molecules over $\gamma$-graphyne (**a**) and $\alpha$-graphyne (**b**). Potential energy map for CH$_4$ above $\alpha$-graphyne without (**c**) and with strain (**d**). The molecule is at a fixed height above the surface ($h=0.5$ Å).

Let us now carefully analyze the gas-monolayer interaction and the adsorption mechanism. Figure 6a,b display the density profile of CH$_4$ molecules along the direction perpendicular to the $\gamma$- and $\alpha$-graphyne surfaces, respectively. The first peak of the density profile indicates the height and thickness of the first CH$_4$ adsorbed layer. We measured a height of *z* $=$ 3.7 Å and a thickness of $\approx$ 4-5 Å for both solids. In general, we find that both the position and thickness are not affected by the solid type or by deformation, as can be verified in Figs. S6–S8 of the Supplementary Information. Indeed, the height of the adsorbed layer depends on the interaction distance between the gas and the C atoms of the monolayer. Thus, it can be compared with the first peak position on the radial distribution function displayed in Figs. S16–S18.

In the cases of $\alpha$- and $\beta$-graphyne, the density profiles show a slight amount of gas at a distance *z* < 2 Å (see Fig. 6b). The presence of CH$_4$ molecules this close to the surface suggests that the molecules are placed on the graphynes’ hexagonal holes. When the strain is applied, the hole is enlarged; consequently, the amount of CH$_4$ in this region increases. This result is critical to understanding the increase of 3.9% in the amount of methane on $\alpha$-graphyne after the strain application. Examining Table 3, we find that the enthalpy of adsorption still decreased in this case, although by a small percentage (15.9%). However, while strain reduced the magnitude of the gas-solid interaction, it also increased the volume available to the gas near the monolayer, as evidenced by Fig. 6b. The outcome of these competing factors was a slight increase in methane adsorption.

In turn, Fig. 6c,d help us understand the increased amount of methane near the monolayer. These images display the interaction energy between CH$_4$ and $\alpha$-graphyne for structures with and without strain (h$=$0.5 Å). In the color scheme used, red (blue) indicates regions where a CH$_4$ molecule would experience repulsive (attractive) interactions with the monolayer. Comparing the two figures, we observe that strain greatly expands the attractive area near the surface at h$=$0.5 Å. Overall, for heights below h<1.5 Å, strain increases the volume where methane-$\alpha$-graphyne interactions are attractive. Inspection of the CH$_4$/$\alpha$-graphyne interaction energy along the axis passing through the center of the hole reinforces this analysis (see Fig. S19c). There, we notice that the deformation removes the energy barrier and turns the center of the hole more attractive for CH$_4$ accommodation. In the Supplementary Information, we also calculate the distribution of molecules near the $\alpha$-graphyne surface for H$_2$, CO$_2$, and CH$_4$ (Figs. S9–S11). These results illuminate why the adsorption of CH$_4$ increases with the strain and those of CO$_2$ and H$_2$ do not.

## Conclusions

In summary, we combined Molecular Dynamics and Grand Canonical Monte Carlo simulations to obtain the mechanical and adsorption properties of graphene, $\alpha$-graphyne, $\beta$-graphyne, and $\gamma$-graphyne under biaxial tensile strain. We found that graphene ($\alpha$-graphyne) has the highest (lowest) ultimate strength and biaxial modulus out of the considered materials. Our calculations also reveal that all monolayers withstand more than 10% biaxial deformation before fracture. Concerning the adsorption properties, we calculated adsorption isotherms for graphene and the graphynes interacting with H$_2$, CO$_2$, and CH$_4$. Results showed that graphene was the 2D solid with the highest adsorption capacity for all gases investigated. We also found that higher capacities were related to higher enthalpies of adsorption and higher surface densities.

Regarding the adsorption on structures under biaxial strain, our results reveal that deformation considerably reduces the amount of gas adsorbed for nearly all gas-solid combinations investigated. This reduction is mainly due to weaker gas-solid interactions in strained monolayers. One exception occurred for $\alpha$-graphyne, in which the quantity of methane adsorbed increased, even though the gas-solid interactions became slightly weaker. However, the reduced adsorption enthalpy was offset by an increased volume available for the gas near the monolayer. Additional calculations revealed that this increase was due to the expansion of regions where the gas-solid interactions are attractive at heights below 1.5 Å above the surface. Finally, our results show that the pore diameter can be controlled through biaxial strain. An analysis comparing pore sizes with kinetic diameters of various gases indicates possible uses of $\alpha$-graphyne (with and without deformation) in gas separation.

## Methods

The MD simulations were performed through the LAMMPS code^65^, using the AIREBO reactive potential^66^ to describe the atomic interactions. The AIREBO potential was designed to describe various hydrocarbon systems^66^ and is commonly used in the literature to describe the mechanical properties of graphyne monolayers^59,67,68^. We also set the cutoff parameter for the REBO part of the potential to 2.0 Å to avoid excessive forces near the fracture of the materials, following previous reports^69,70^. The simulated 2D structures are illustrated in Fig. 1 and have the following dimensions: 37 Å $\times$ 28 Å for $\alpha$-graphyne; 29 Å $\times$ 33 Å for $\beta$-graphyne; 27 Å $\times$ 25 Å for $\gamma$-graphyne; and 25 Å $\times$ 24 Å for graphene. These values correspond to the sheet dimensions after geometry optimization obtained from energy minimization applying the conjugate gradient algorithm^65^. A vacuum layer of at least 40 Å was introduced to separate periodic images of the system and a time step of 0.1 fs was used during the MD simulations.

Regarding the deformation process, it was divided into three parts:

1. We initialized all atoms at a temperature of 298 K and then thermalized the system for $1.0 \times 10^6$ steps at 298 K and 0 atm. A chain of three Nose–Hoover thermostats and barostats was used to control the temperature and pressure^71^.
2. We turned off the thermostat and barostat and thermalized the system for an additional $1.0 \times 10^5$ steps in the NVE ensemble. We also used this ensemble during the next part of the deformation process.
3. We applied biaxial strain to the material at a rate of $10^{-6}$ per femtosecond until it fractured. Strain and stress values were recorded while the deformation occurred. To compute the stress in GPa, we assumed a thickness of 3.4 Å for all the considered monolayers.

For the GCMC simulations, we employed the RASPA molecular simulation software^72^ to obtain the gas adsorption isotherms curves for structures with and without strain. During the GCMC simulations, the monolayer atoms are fixed and have zero partial charges; then, the host–gas interactions are described only by the Lennard–Jones potential with parameters extracted from the Universal Force Field^73^. We discuss in more detail these methodology choices in the Supplementary Information. This approach has largely been used to study physical adsorption on solid materials, and the accuracy of generic force fields usually depends on the system studied. For the purpose of this work, the choice of the force field might affect the quantitative results but should not interfere with its main conclusions.

The CO$_2$ molecule was represented by the conventional rigid linear triatomic model, with the three charged and LJ interaction sites located on each atom, as previously derived by Harris and Yung^74^. The CH$_4$ molecule was described by the TraPPE uncharged single LJ interacting site model^75^. The H$_2$ molecules were modeled with uncharged two-sites LJ^76^. The cutoff of 14.0 Å for both the Lennard–Jones and Coulomb interactions were applied and Ewald summation was used for the calculations of electrostatic long-range interactions. The GCMC simulations were performed considering the probabilities for three types of Monte Carlo moves, which are 20% of translation, 20% of rotation, and 60% of swaps (insertion and removal). These probability values do not affect the results. In Fig. S29 of the Supplementary Information, we test different sets of parameters for the case of CO$_2$ molecules adsorbing in graphene at 50 atm and 298 K. For CO$_2$ and CH$_4$, we obtained adsorption isotherms for a temperature of 298 K and employed pressure values ranging from $1.0 \times 10^3$ to $2.0 \times 10^5$ Pa. For H$_2$, we considered the same pressure range, but a lower temperature of 77 K. We selected T = 77 K in this case because the amount of gas adsorbed was low at higher temperatures. We used 10$^4$ simulation cycles to initialize the simulation and then 10$^4$ more cycles to obtain the adsorption data. The average adsorption energy $\Delta U$ is calculated using the Widom insertion method^72,77,78^ and the enthalpy of adsorption at infinite dilution is given by $\Delta H=\Delta U-RT$, where R is the universal gas constant and T the temperature^79^.

For post-simulations analyses we obtained the density profile along *z*-direction by computing the histograms of the height position of the adsorbed molecules. The histograms were constructed using bin width equal 1 Å and slider width equal 0.1 Å. To increase the number of adsorbed molecules and improve the statistics, we performed GCMC simulations at a higher pressure of 50 bar for the histogram calculations. We also mapped the interaction between a gas molecule at a given height and a monolayer using the LAMMPS code. To achieve this, we fixed the z position of a gas molecule and varied its x and y positions to scan the surface of the 2D material. And we recorded the interaction energy as we changed the molecule’s position. For each interaction energy map, we scanned an area with dimensions 20 Å $\times$ 20 Å and, for both planar directions, we displaced the molecules using 0.1 Å increments (for a total of $4.0 \times 10^4$ single point energy calculations).

## Supplementary Information

Supplementary Information.

## Data Availability

The datasets used and/or analysed during the current study available from the corresponding author on reasonable request.
