# Supplementary material for: Mechanical and gas adsorption properties of graphene and graphynes under biaxial strain
Source: Sci Rep. 2022 Dec 27;12:22393. doi: 10.1038/s41598-022-27069-y (PMC9794739; doi:10.1038/s41598-022-27069-y)
Supplement: Supplementary file 1 — Supplementary Information. [file 41598_2022_27069_MOESM1_ESM.pdf]

Supplementary Information: Mechanical and gas adsorption properties of graphene and graphynes under biaxial strain

Raphael B. Oliveira,[1] Daiane Damasceno Borges,[2] and Leonardo D. Machado[1,*]

[1]*Departamento de Física, Universidade Federal do Rio Grande do Norte, 59072-970, Natal, RN, Brazil.*

[2]*Physics Institute, Federal University of Uberlandia, Uberlandia-MG, 38408-100, Brazil.*

(Dated: December 26, 2022)

CONTENTS

Mechanical properties at T = 10 K 3

Grand Canonical Monte Carlo simulations: detailing some methodological choices 5

Adsorption isotherms for H_2 and CH_4 6

Histogram results: Analyzing the distribution of molecules over the monolayers. 10

Molecule distribution maps for α-graphyne: Considering molecules located between 0 and 2 Å. 13

Molecule distribution maps for all monolayers: Considering molecules located between 2 and 5.5 Å. 16

Radial Distribution Function results 21

Potential energy results: moving molecules through the graphyne pores 24

Potential energy results: comparing DFT and UFF results 28

Potential energy maps for H_2 and CO_2 near the surface of α-graphyne 31

Considering the effect of domain size on the adsorption properties: changing the size of the monolayer unit cell 33

Testing convergence considering different probabilities for the Monte Carlo moves. 35

References 36

MECHANICAL PROPERTIES AT T $= 10$ K

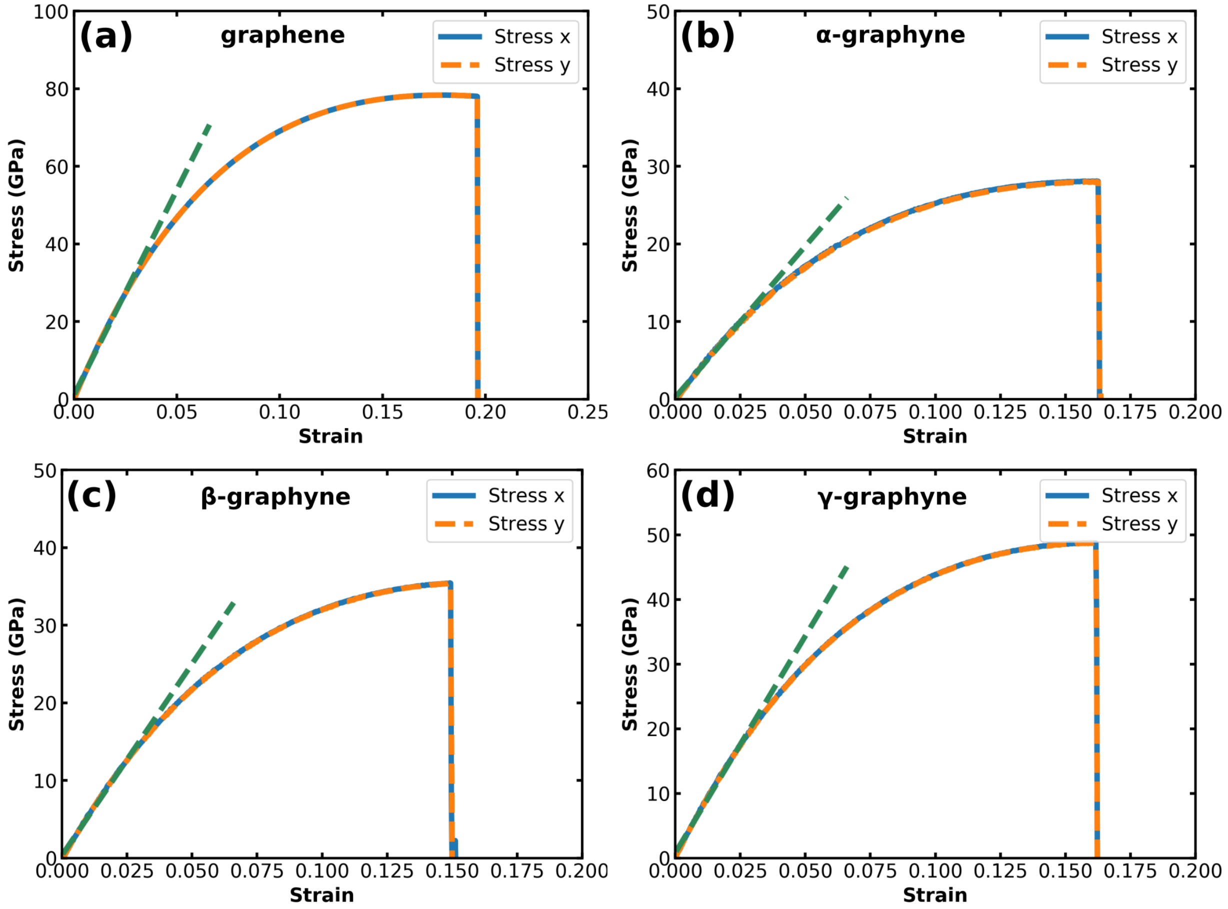

FIG. S1. This figure displays stress-strain curves for graphene and the three graphynes considered at T$=$ 10 K. We present both stress values along the x and the y directions in this figure. The biaxial modulus, ultimate strain, and ultimate strength feature higher values than those obtained at T $=$ 298 K. The results obtained for γ-graphyne at T$=$ 10 K indicate reasonable agreement between our MD results and data from previous DFT calculations at T$=$ 0 K. For comparison, Peng *et al.* obtained for γ-graphyne under biaxial strain an ultimate strain of 0.18 and an ultimate strength of 60.7 GPa [1]. Results were converted from N/m, assuming a thickness of 3.4 Å for the monolayers.

TABLE I. Mechanical properties of graphene and the graphynes under biaxial strain at 10 K. Results were converted from N/m assuming a thickness of 3.4 Å for the monolayers.

	Biaxial modulus (GPa)	Ultimate strain	Ultimate strength (GPa)
Graphene (this work)	1058	0.181	78.4
α-graphyne (this work)	389.7	0.162	28.1
β-graphyne (this work)	493.2	0.149	35.5
γ-graphyne (this work)	670.7	0.160	48.8

GRAND CANONICAL MONTE CARLO SIMULATIONS: DETAILING SOME METHODOLOGICAL CHOICES

In this work, we follow other articles in the literature that use Grand-Canonical Monte Carlo simulations to reproduce experimental gas adsorption isotherms using only a generic force field to describe the Lennard Jones interactions and partial charges to describe the Coulomb interactions [2]. This approach, which does not consider any flexibility of adsorbents/adsorbates, is frequently adopted to mimic the adsorption of small molecules on solid materials that occur mainly through van der Waals forces. In particular, the gas adsorption on 2D carbon materials should not provoke any important movements, such as folds or wrinkles in the sheet. The isotherms are not strongly impacted by the flexibility as shown in previous work [3]. In addition, in our work, we are interested in studying adsorption under strain, which would limit even more the movement of the sheet during adsorption and, thus, justifies using a rigid body approach to mimic the carbon monolayer.

Concerning the fact that partial charges carried by carbon atoms of graphene/graphynes are set to zero. We note that an infinite neutral pristine graphene has neither boundary effects nor dopants with different chemical groups that justify having an inhomogeneous charge distribution. Thus, we are convinced that the gas-molecule interactions for an infinite neutral pristine graphene/graphyne can be described only by the Lennard Jones potential, as has been done in other molecular simulations found in the literature [2]. To support this statement, Wang *et. al.* [4] have shown through DFT calculations that CO_2 physisorption on pristine graphene occurs through van der Waals forces, with little charge transfer.

ADSORPTION ISOTHERMS FOR H_2 AND CH_4

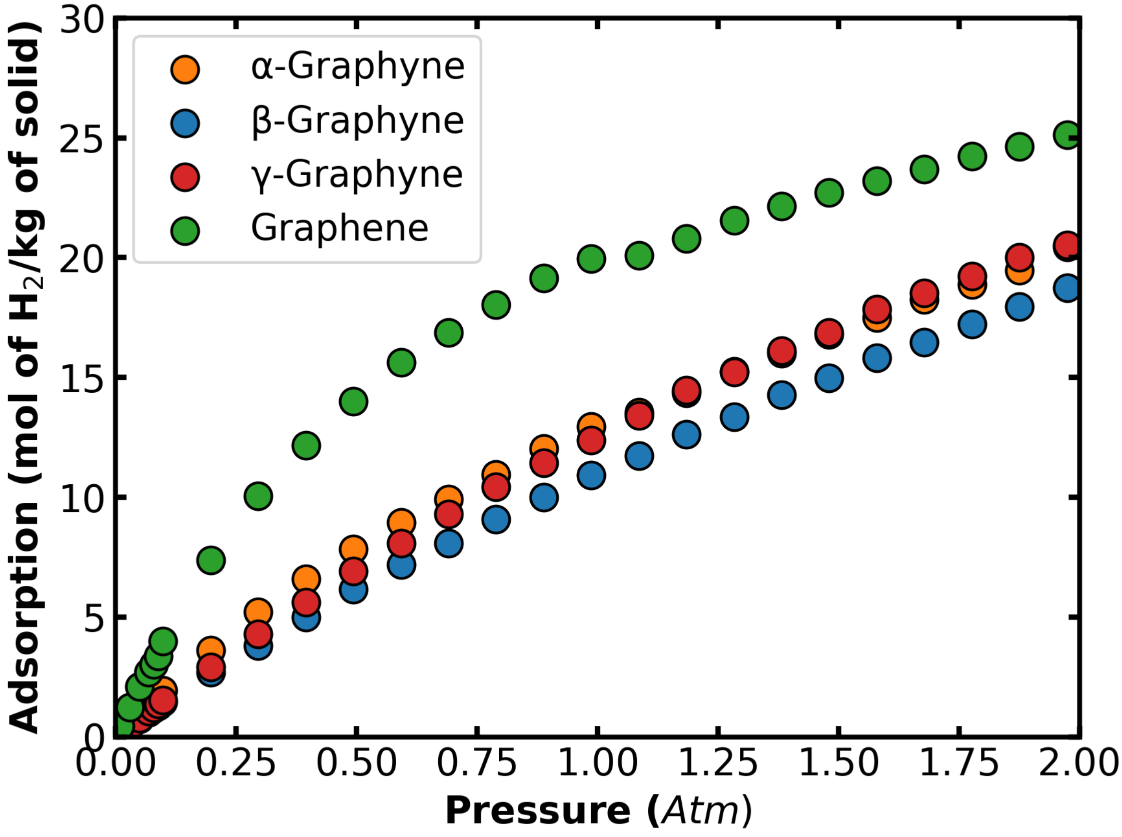

FIG. S2. Adsorption isotherms of H_2 on graphene and the three graphynes without strain (T = 77 K). Due to the low temperature, we were able to observe a greater amount of adsorbed gas

This section displays the adsorption isotherms omitted from the main text. Note that results for H_2 were obtained at 77 K, as the amount of H_2 adsorbed on all considered monolayers was low at T = 298 K.

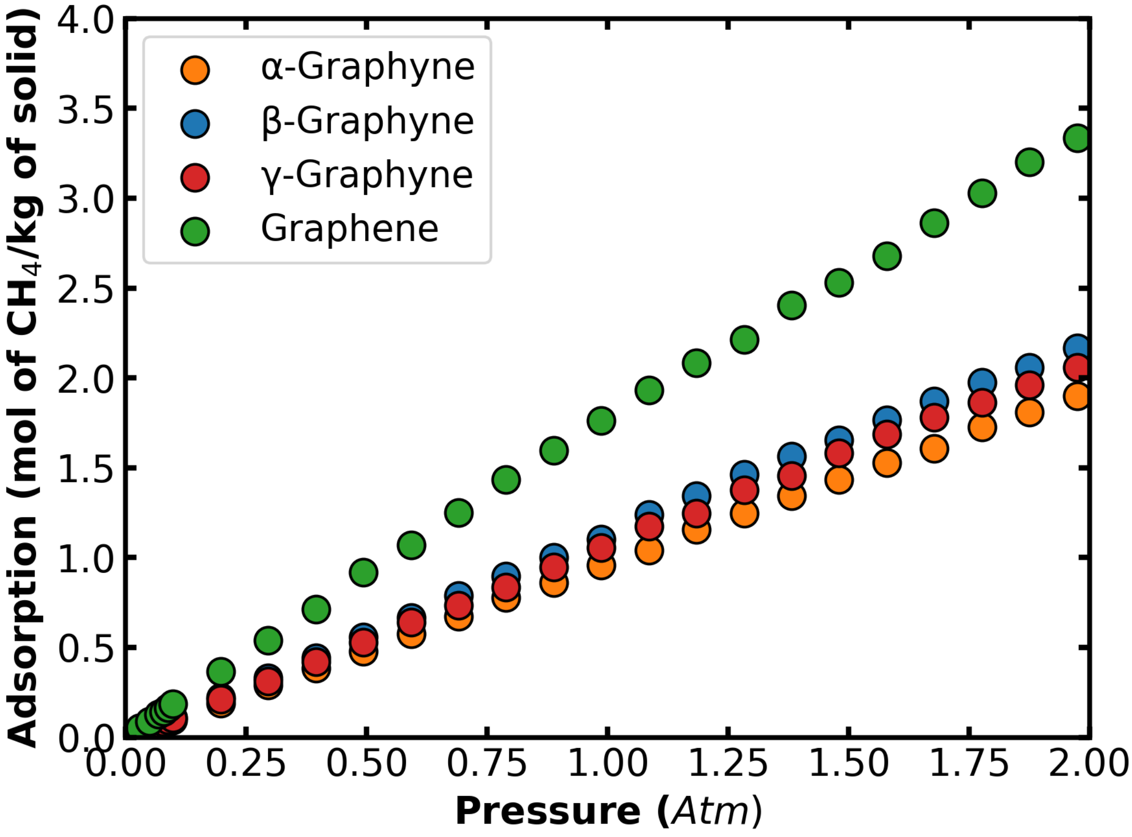

FIG. S3. Adsorption isotherms of CH_4 on graphene and the three graphynes without strain (T = 298 K). For methane, the amount of gas adsorbed on the graphynes is similar.

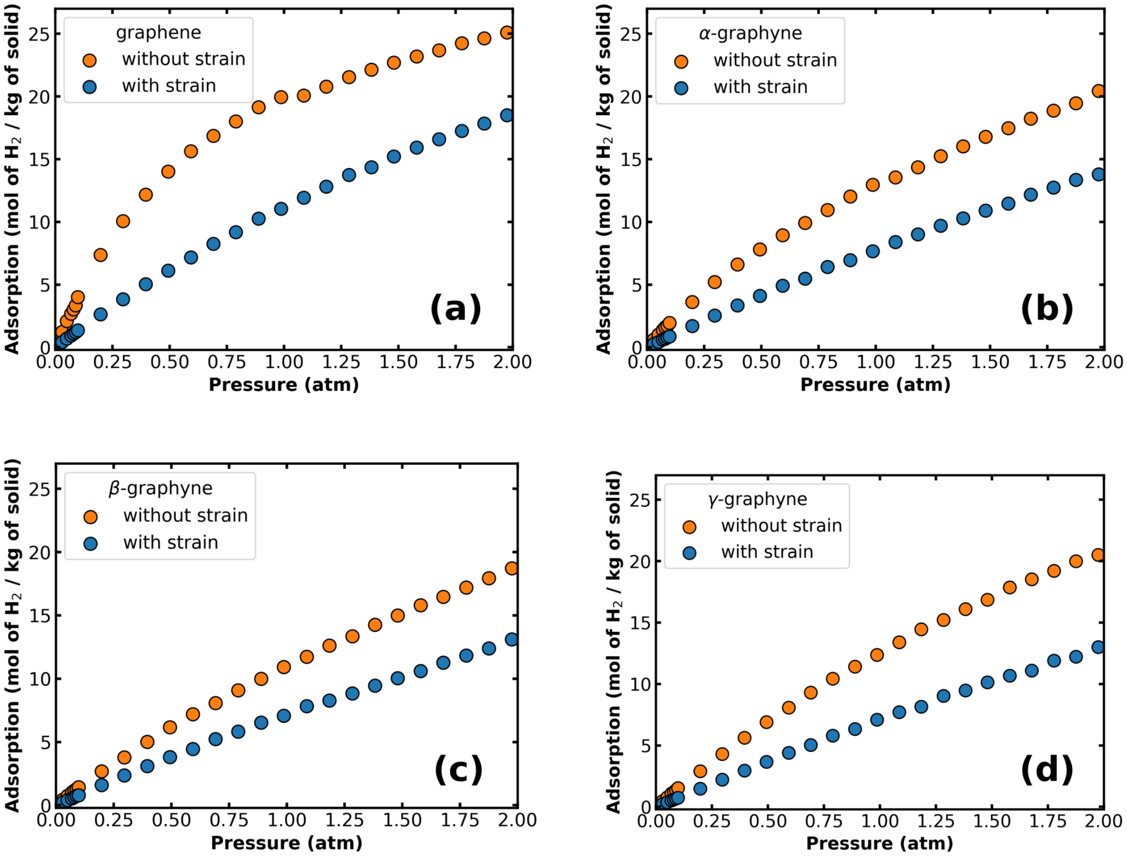

FIG. S4. Adsorption isotherms of H_2 at T = 77 K, comparing results with and without strain.

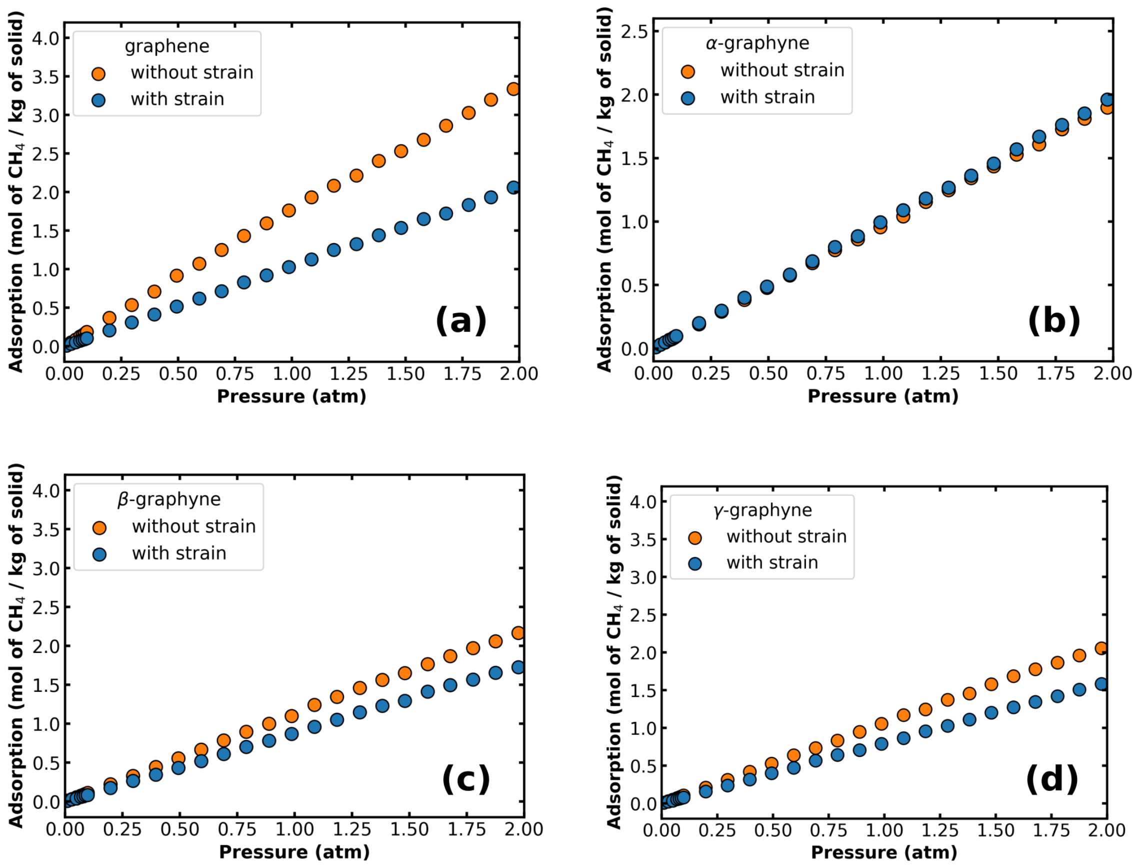

FIG. S5. Adsorption isotherms of CH_4 at T = 298 K, comparing results with and without strain.

HISTOGRAM RESULTS: ANALYZING THE DISTRIBUTION OF MOLECULES OVER THE MONOLAYERS.

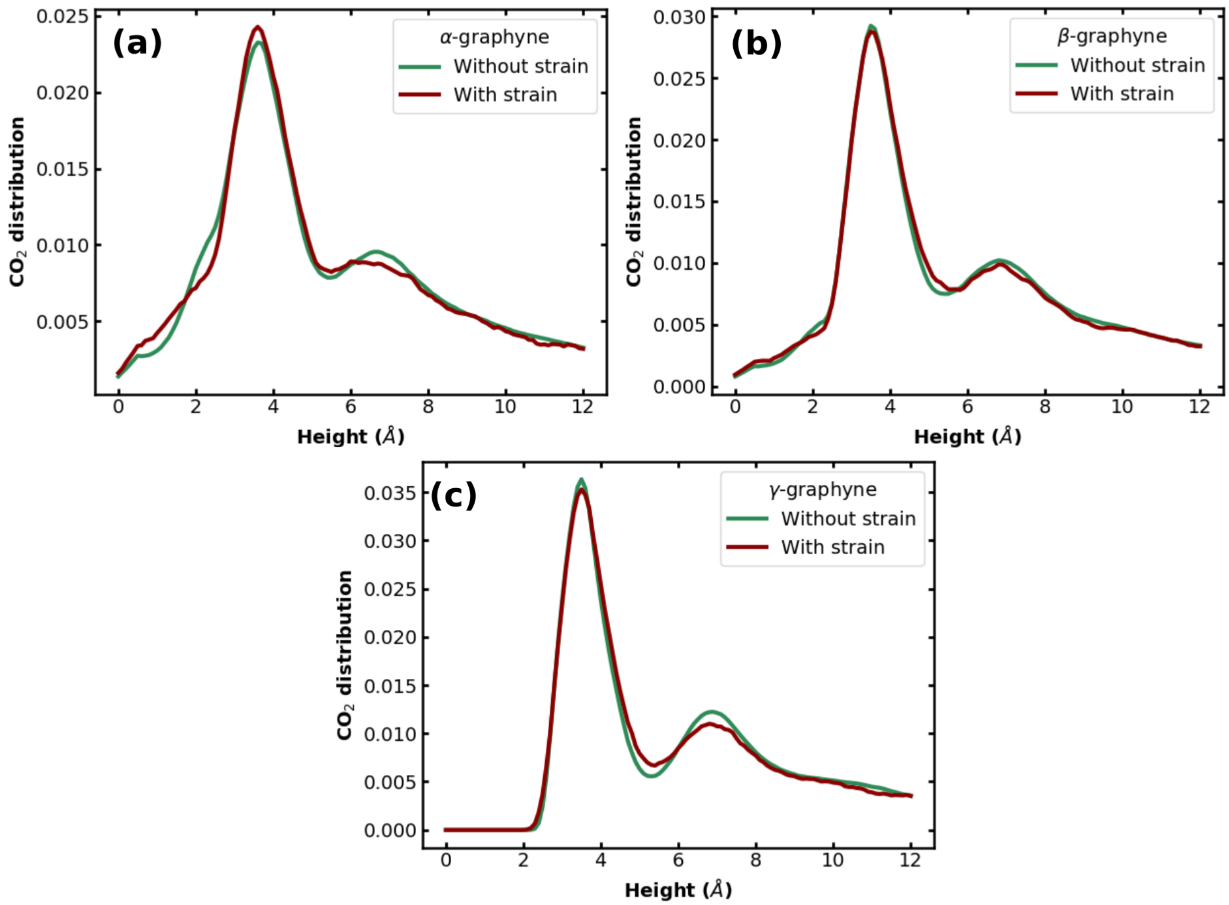

FIG. S6. Density profile of CO_2 molecules over the graphynes under thermodynamical conditions of P = 50 atm and T = 298 K. The position of the first peak does not change with sheet deformation. The positions are 3.6 Å, 3.5 Å and 3.5 Å for α-, β- and γ-graphyne, respectively.

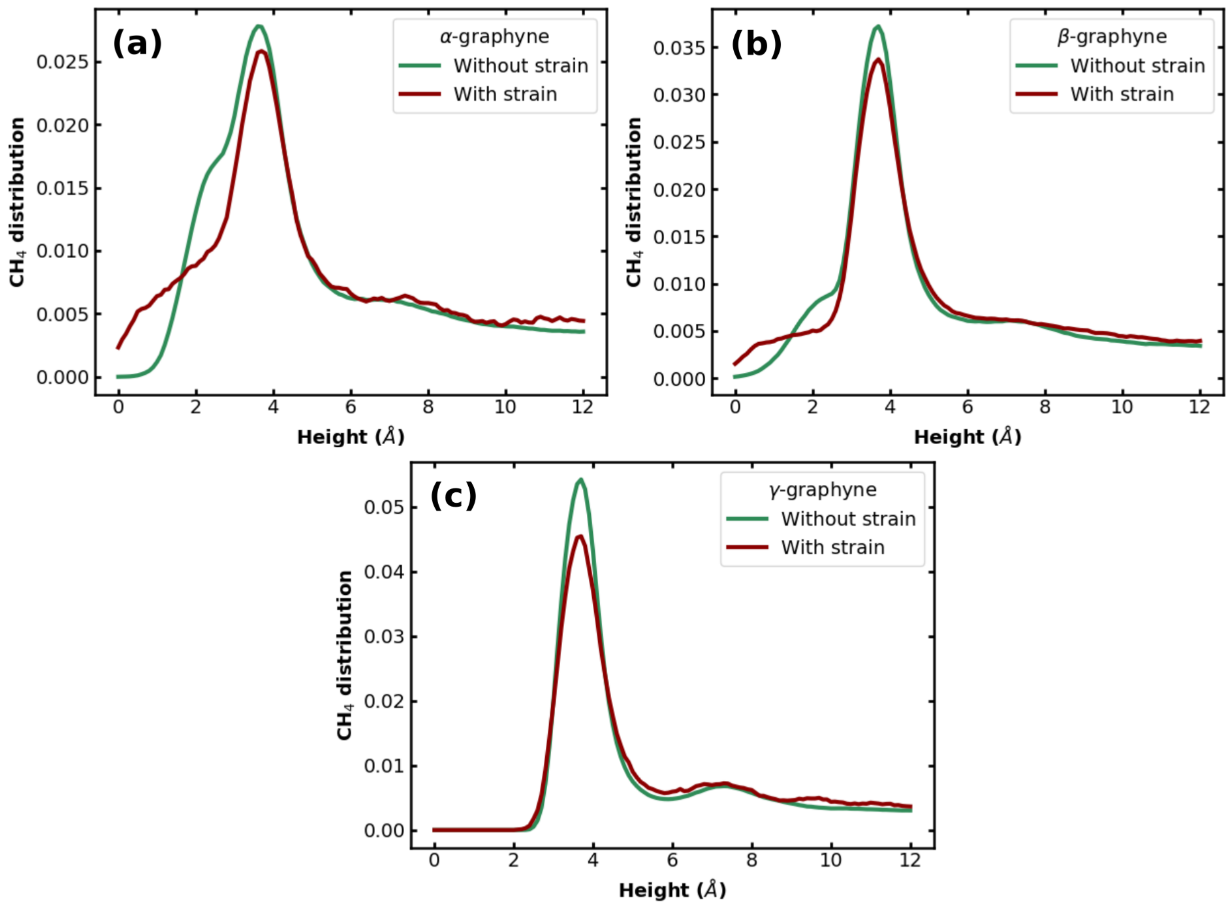

FIG. S7. Density profile of CH_4 molecules over the graphynes under thermodynamical conditions of P = 50 atm and T = 298 K. The position of the first peak does not change with sheet deformation, except a slightly difference for α-graphyne. The positions are 3.6 Å/3.7Å (without/with strain), 3.7 Å and 3.7 Å for α-, β- and γ-graphyne, respectively.

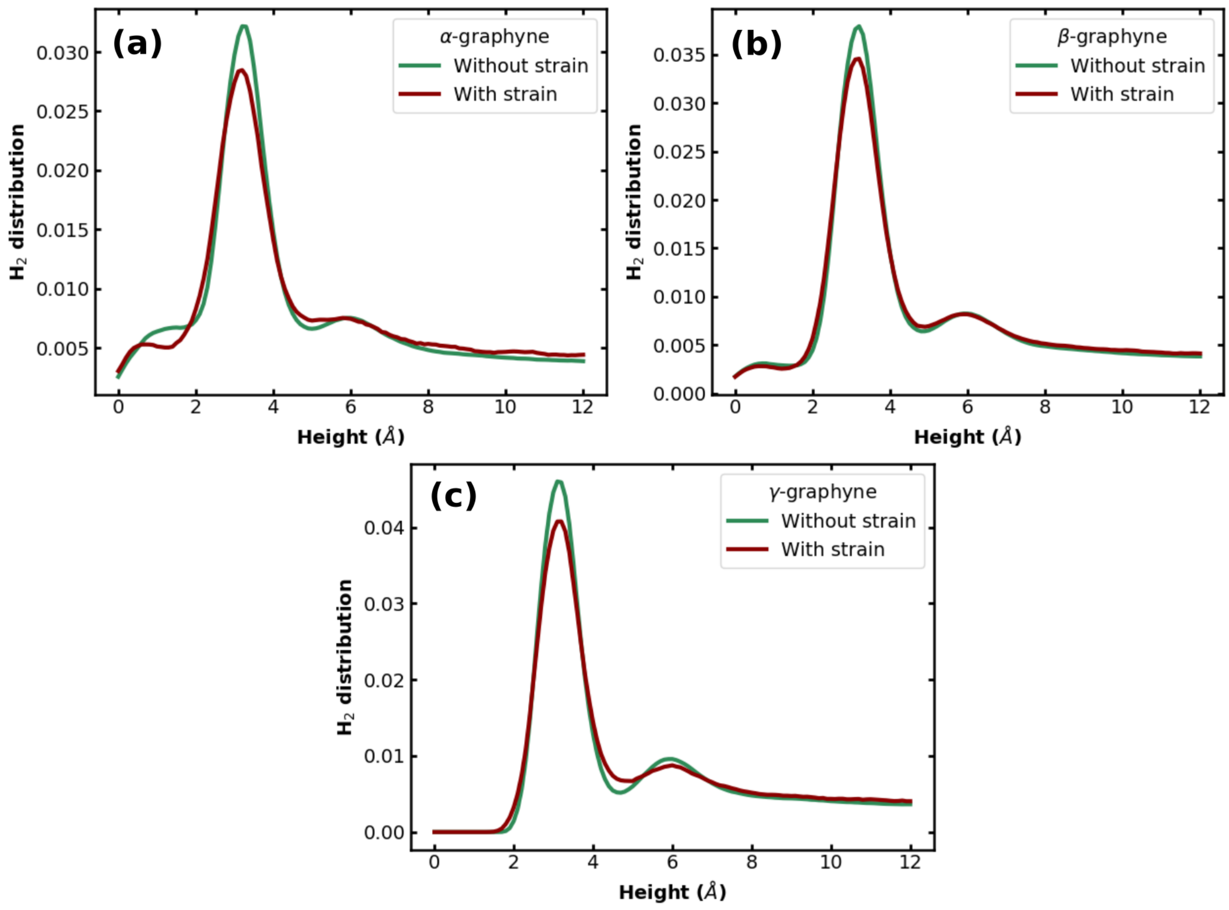

FIG. S8. Density profile of H_2 molecules over the graphynes under thermodynamical conditions of P = 50 atm and T = 77 K. The position of the first peak does not change with sheet deformation, except a slightly difference for γ-graphyne. The positions are 3.2 Å, 3.2 Å and 3.1 Å/3.2 Å (without/with strain) for α-, β- and γ-graphyne, respectively.

MOLECULE DISTRIBUTION MAPS FOR α-GRAPHYNE: CONSIDERING MOLECULES LOCATED BETWEEN 0 AND 2 Å.

We used the Gaussian kernel density estimation method implemented in the scipy python library to calculate molecular distribution maps. In this method, a gaussian kernel is placed on each data point, and then the kernels are added to estimate the probability density function for the position of the molecules.

We implemented this method for a 2D array of data points, allowing us to plot 2D molecule distribution maps. To obtain these maps, we selected a range for the heights above the monolayer plane and then collected the x and y positions for all molecules within this range. This procedure allowed us to determine where in the plane the molecules are placed for the given range in height.

It is important to notice that the Gaussian kernel density estimation method does not consider the system's periodicity, even though the simulations were carried out using periodic unit cells. Hence, regions near the boundaries have fewer data points in their vicinity and tend to appear as regions with lower molecule density in the maps.

To collect the data points, we stored the positions of all molecules that were adsorbed in the monolayers. We performed 10000 GCMC cycles and saved the molecular positions every ten cycles. Note that during the simulations, molecules may move above the surface, may desorb from the monolayer, or new molecules may adsorb on the material.

These results help us understand why the adsorption of CH_4 in α-graphyne increases with the strain and those of H_2 and CO_2 do not. This difference is related to the size of the molecules. Since methane is the gas with the largest kinetic diameter, without applying strain, it is difficult for the CH_4 molecules to access the sites near the α-graphyne plane. Comparing the three cases, we find that volume accessed by the gas increased considerably more for the case of methane. Furthermore, we find that, without strain, a few pores of α-graphyne were never occupied with CH_4.

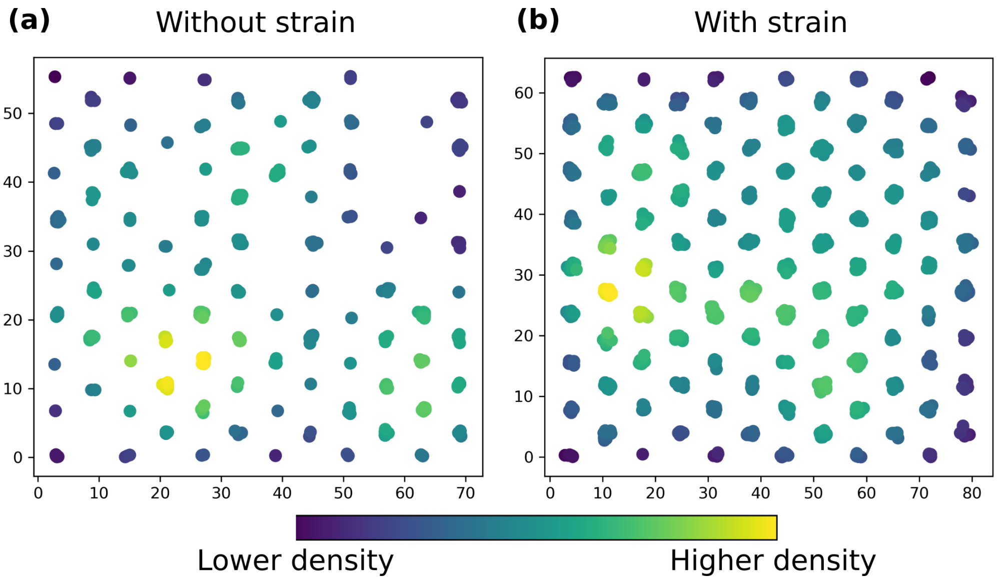

FIG. S9. Distribution maps for CH_4 molecules located at heights between 0 Å and 2 Å above α-graphyne. Close to the monolayer surface, only regions near the pores are occupied. Dimensions in the x− and y− directions are given in Angstroms.

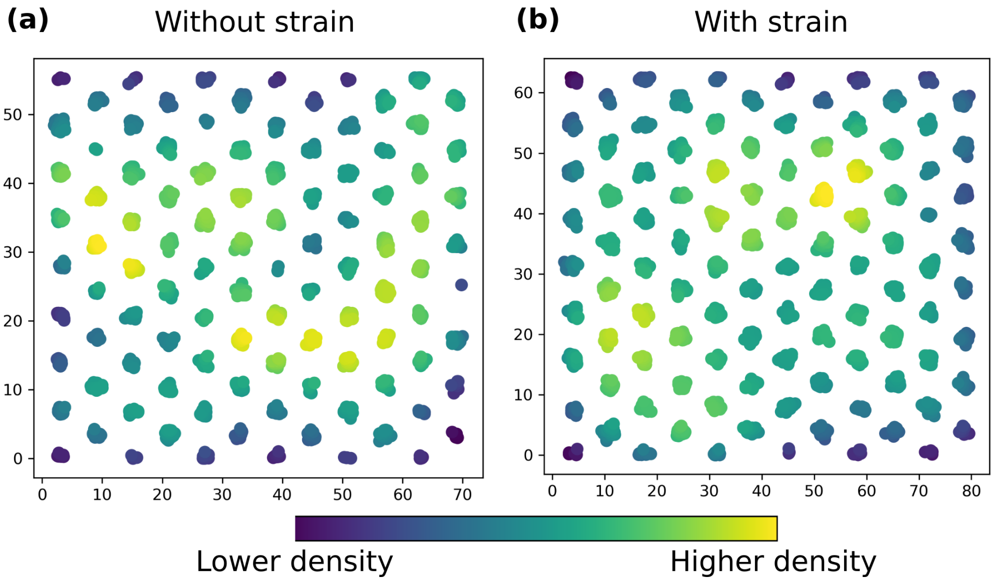

FIG. S10. Distribution maps for CO_2 molecules located at heights between 0 Å and 2 Å above α-graphyne. Close to the monolayer surface, only regions near the pores are occupied. Dimensions in the x− and y− directions are given in Angstroms.

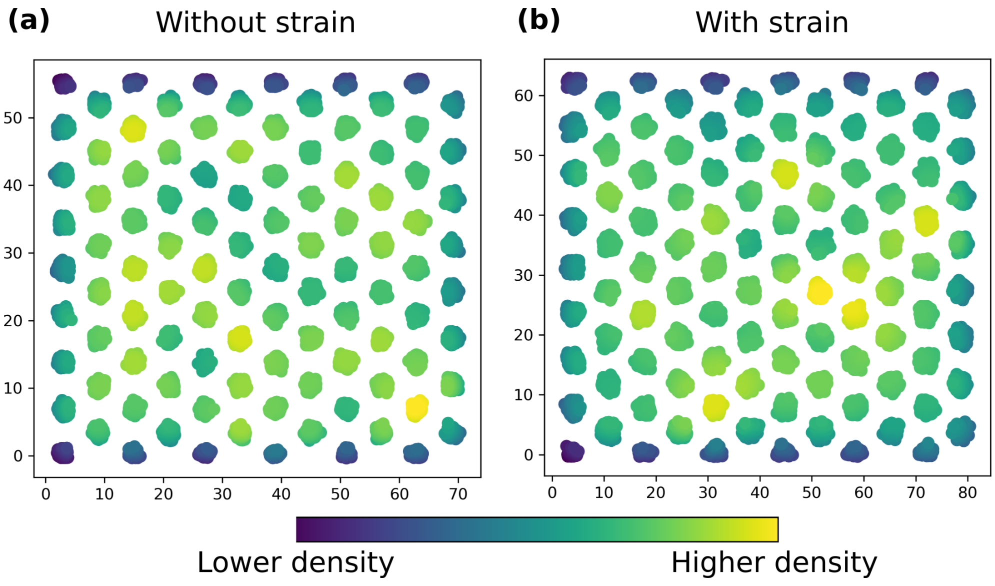

FIG. S11. Distribution maps for H_2 molecules located at heights between 0 Å and 2 Å above α-graphyne. Since the hydrogen molecules are smaller, only the regions near the atoms aren't occupied, even near the monolayer. Dimensions in the x− and y− directions are given in Angstroms.

MOLECULE DISTRIBUTION MAPS FOR ALL MONOLAYERS: CONSIDERING MOLECULES LOCATED BETWEEN 2 AND 5.5 Å.

In this section, we display distribution maps for molecules located between 2 and 5.5 Å above a monolayer surface. We selected this range because the distribution peaks in Figs. S7, S6, and S8 are within these limits.

And once again, we note that the Gaussian kernel density estimation method does not consider the system's periodicity, even though the simulations were carried out using periodic unit cells. Hence, regions near the boundaries have fewer data points in their vicinity and tend to appear as regions with lower molecule density in the maps.

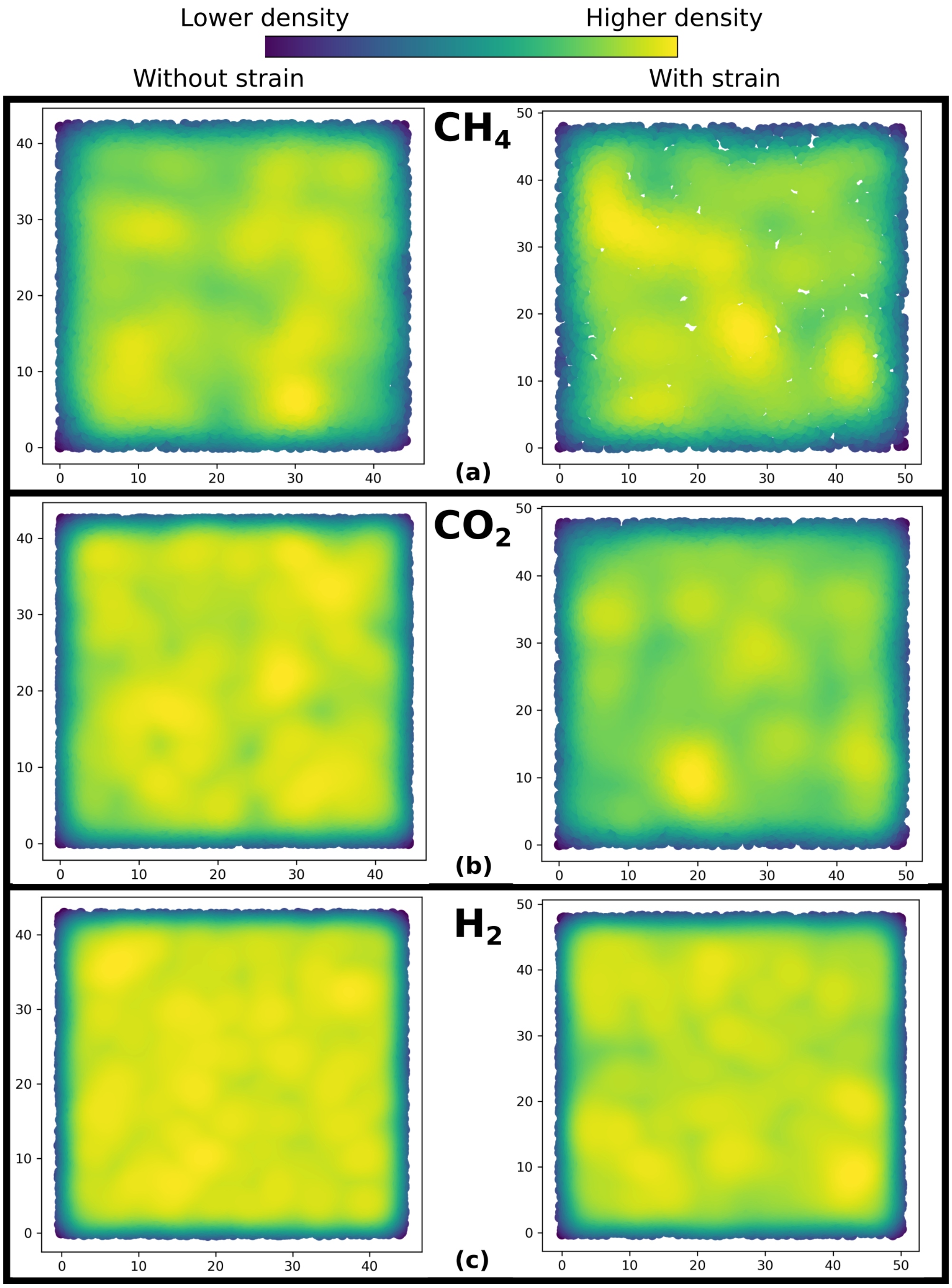

FIG. S12. Distribution maps for gas molecules located at heights between 2.0 Å and 5.5 Å above graphene. Dimensions in the x− and y− directions are given in Angstroms.

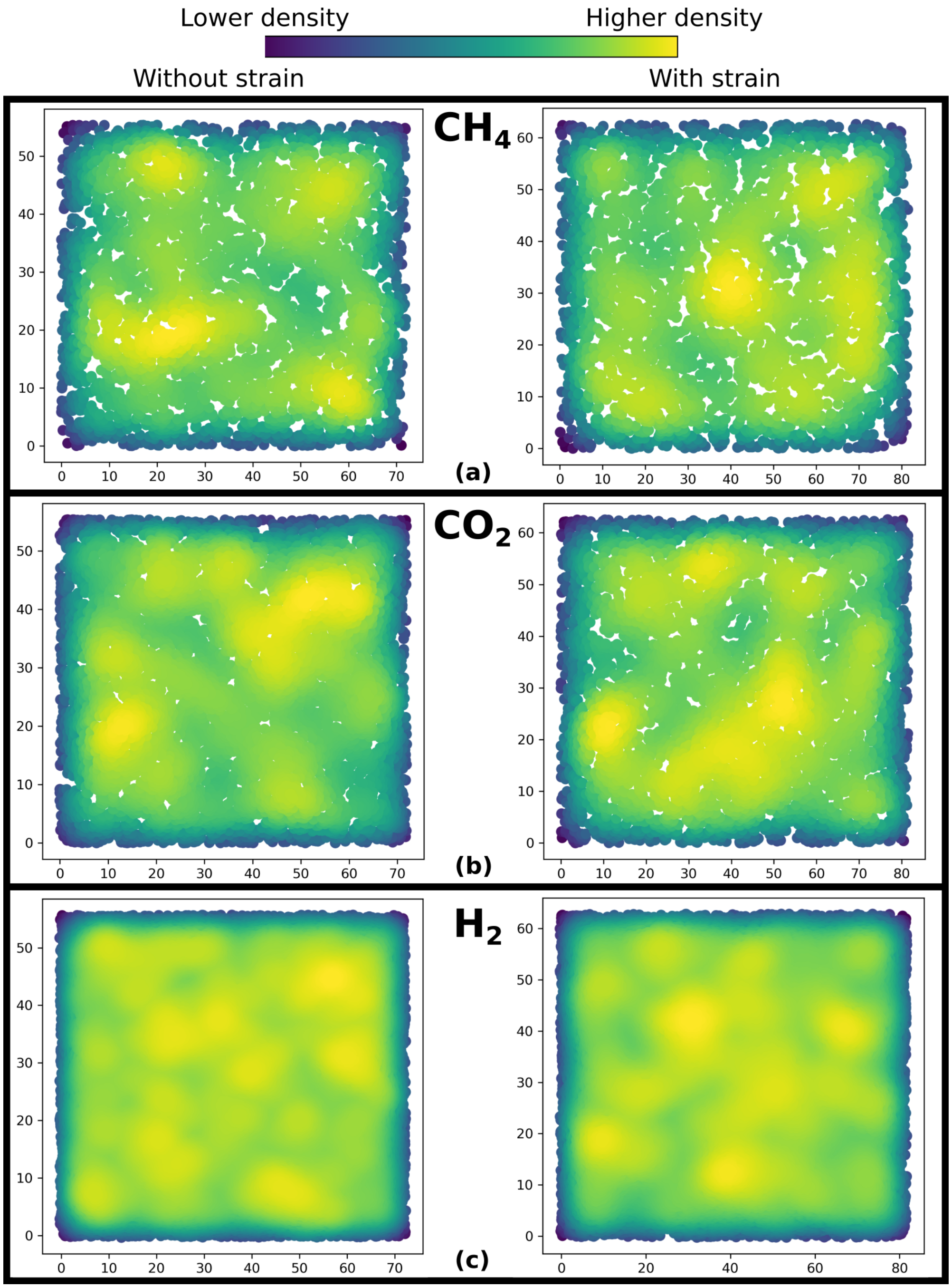

FIG. S13. Distribution maps for gas molecules located at heights between 2.0 Å and 5.5 Å above α-graphyne. Dimensions in the x− and y− directions are given in Angstroms.

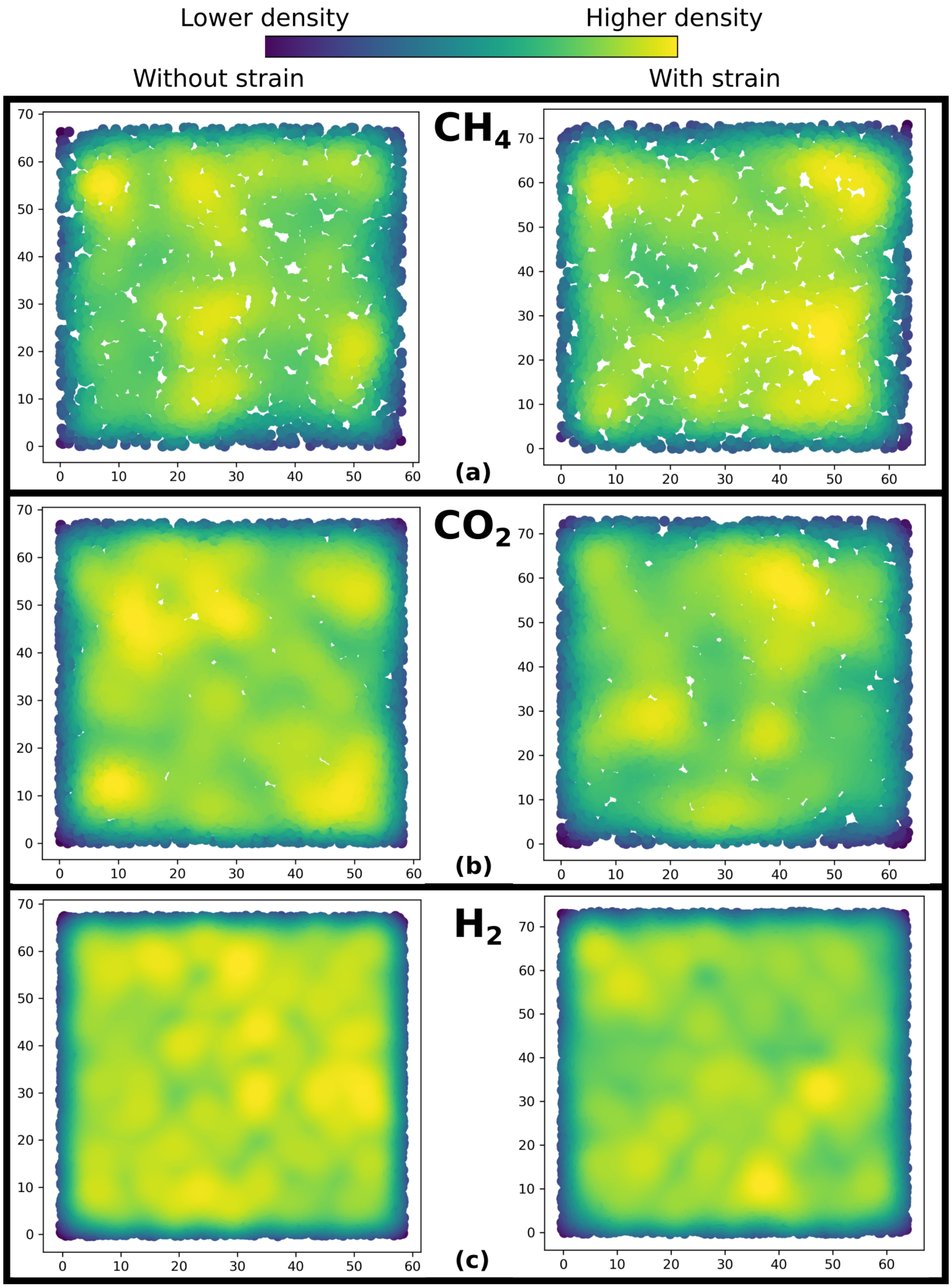

FIG. S14. Distribution maps for gas molecules located at heights between 2.0 Å and 5.5 Å above β-graphyne. Dimensions in the x− and y− directions are given in Angstroms.

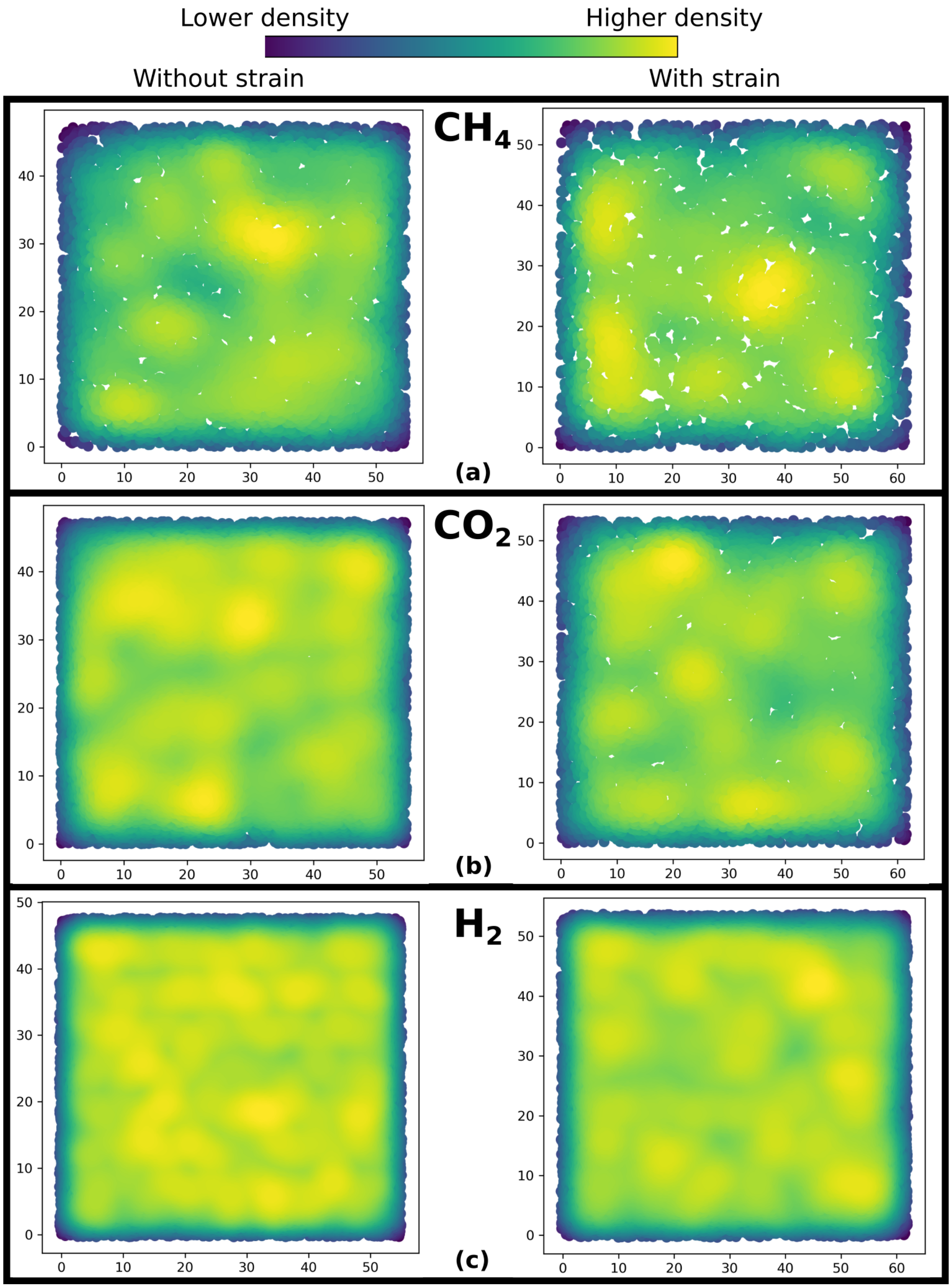

FIG. S15. Distribution maps for gas molecules located at heights between 2.0 Å and 5.5 Å above γ-graphyne. Dimensions in the x− and y− directions are given in Angstroms.

RADIAL DISTRIBUTION FUNCTION RESULTS

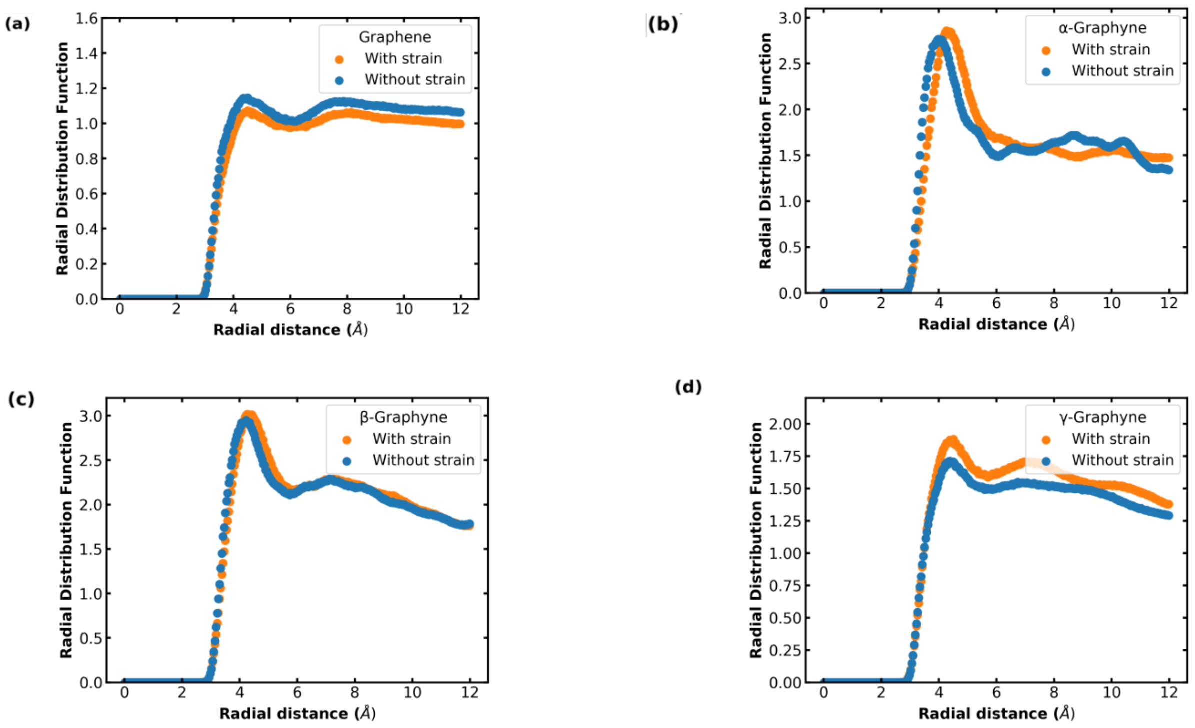

FIG. S16. RDF between C_{CO_2} and C_g of graphene and the graphynes. We used a pressure of 50 atm and a temperature of 298 K. The position of the first peak is 4.5 Å for graphene and γ-graphyne and 4.0/4.3 Å for α- β-graphyne without/with strain.

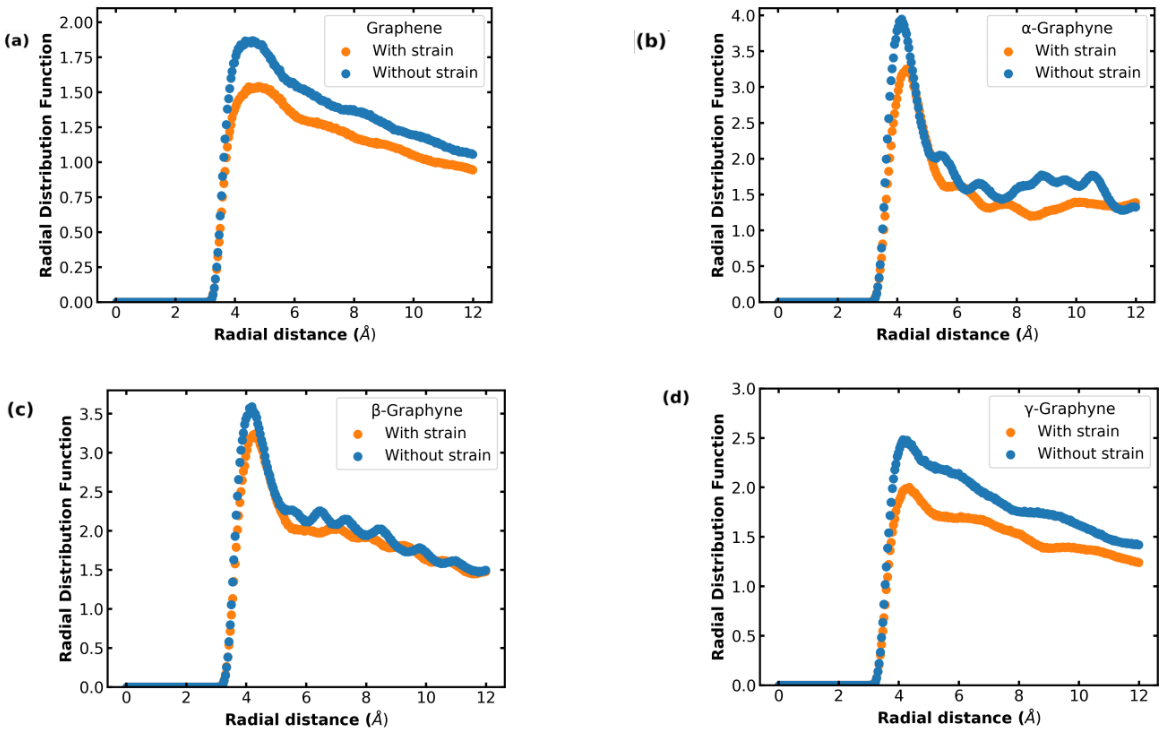

FIG. S17. RDF between C_{CH_4} and C_g of graphene and the graphynes. We used a pressure of 50 atm and a temperature of 298 K. The position of the first peak is 4.5 Å for graphene and γ-graphyne and 4.2/4.3 Å for α- β-graphyne without/with strain.

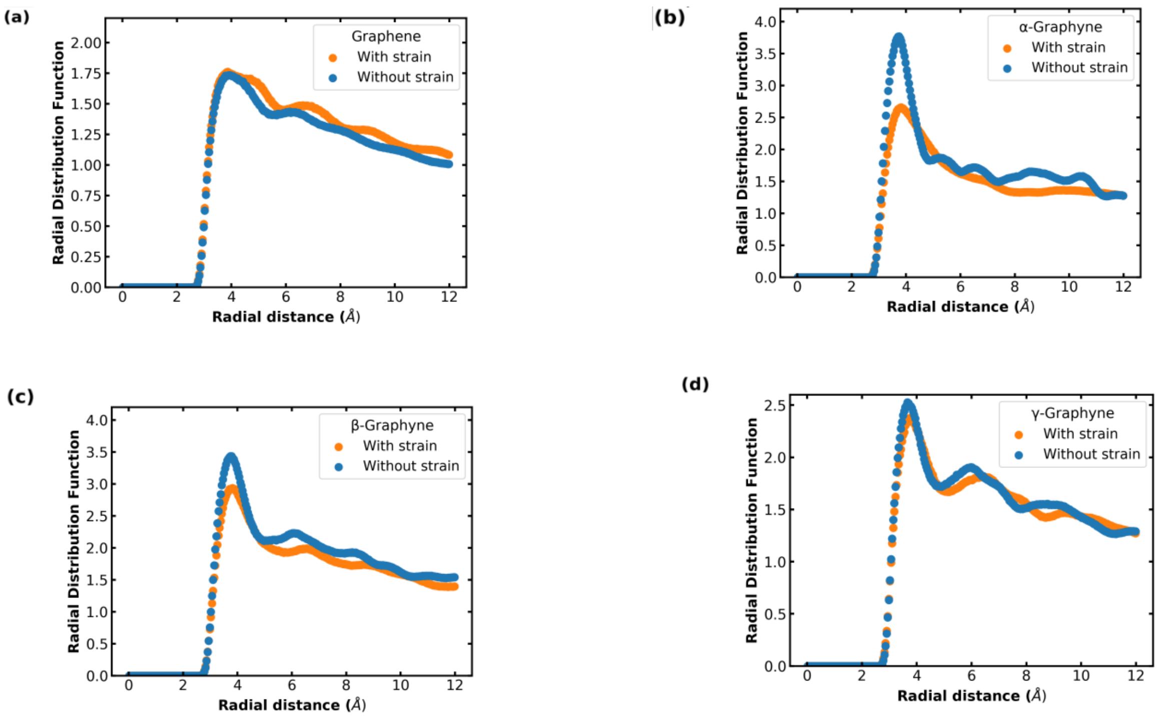

FIG. S18. RDF of H_2 on graphene and the graphynes. We used a pressure of 50 atm and a temperature of 77 K. The position of the first peak is 3.9 Å for graphene and 3.8 Å for the graphynes.

POTENTIAL ENERGY RESULTS: MOVING MOLECULES THROUGH THE GRAPHYNE PORES

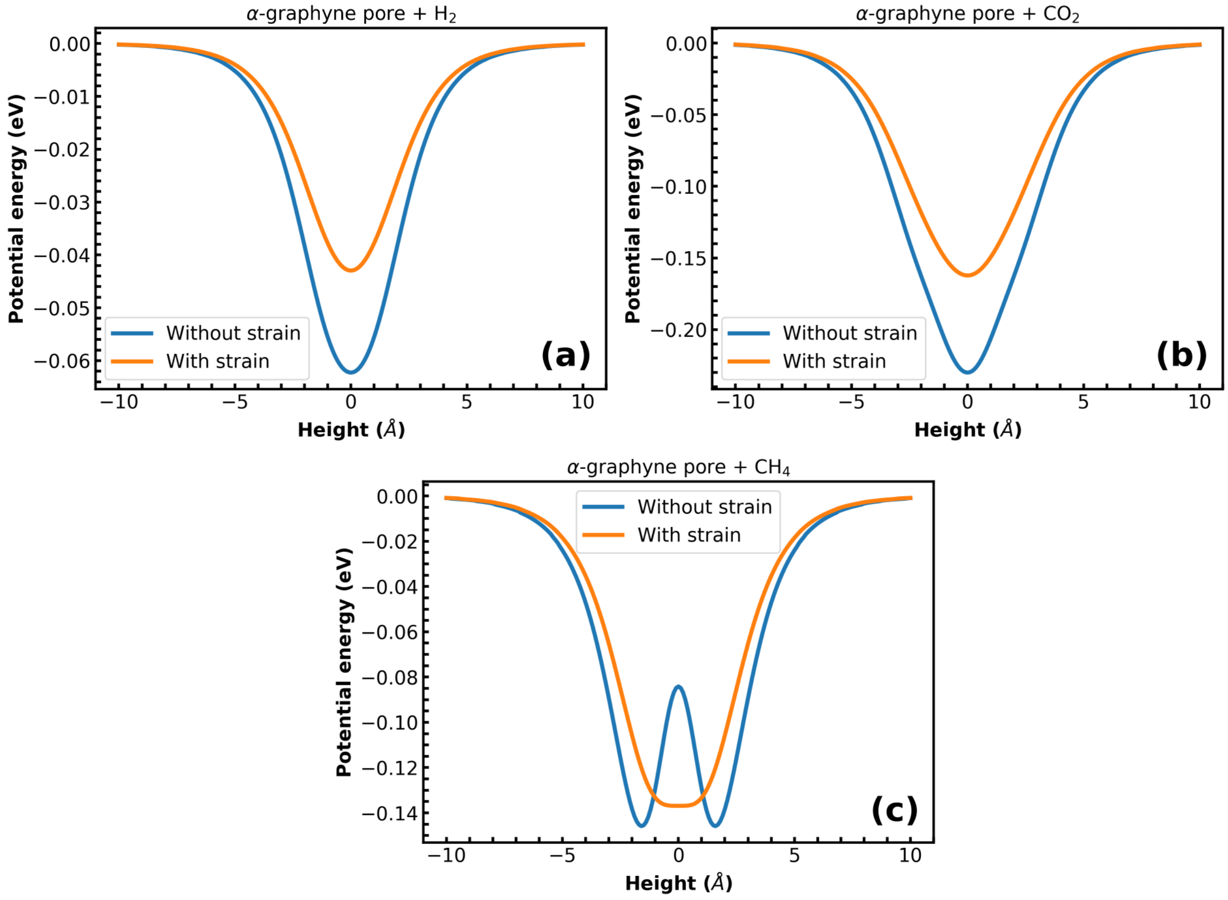

FIG. S19. Potential energy curves obtained by displacing a gas molecule through the center of the pore of α-graphyne. For CO_2 and H_2, the molecule was aligned perpendicular to the plane of the monolayer. The gas-surface interactions were described using UFF for the monolayers and TraPPE for the gas molecules.

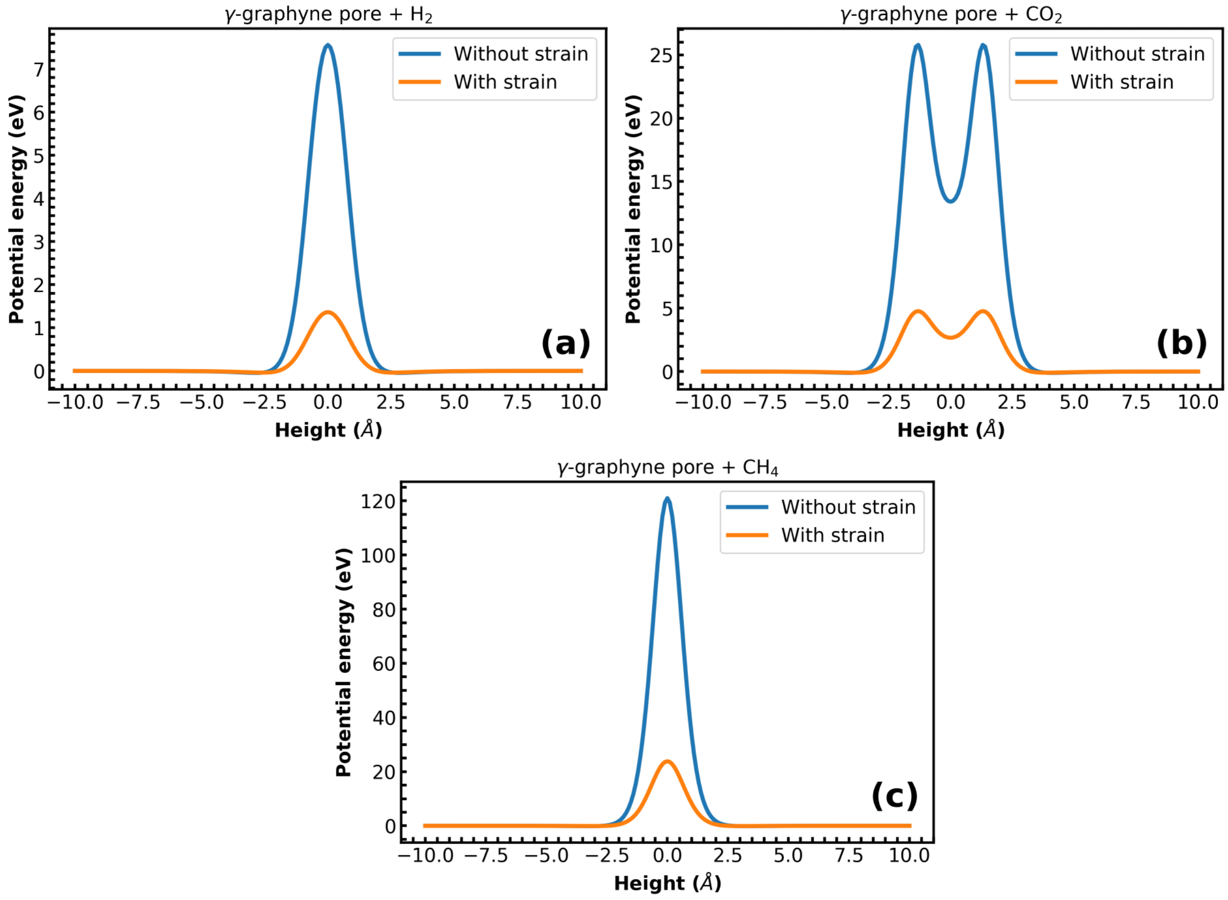

FIG. S20. Potential energy curves obtained by displacing a gas molecule through the center of the pore of γ-graphyne. For CO_2 and H_2, the molecule was aligned perpendicular to the plane of the monolayer. The gas-surface interactions were described using UFF for the monolayers and TraPPE for the gas molecules.

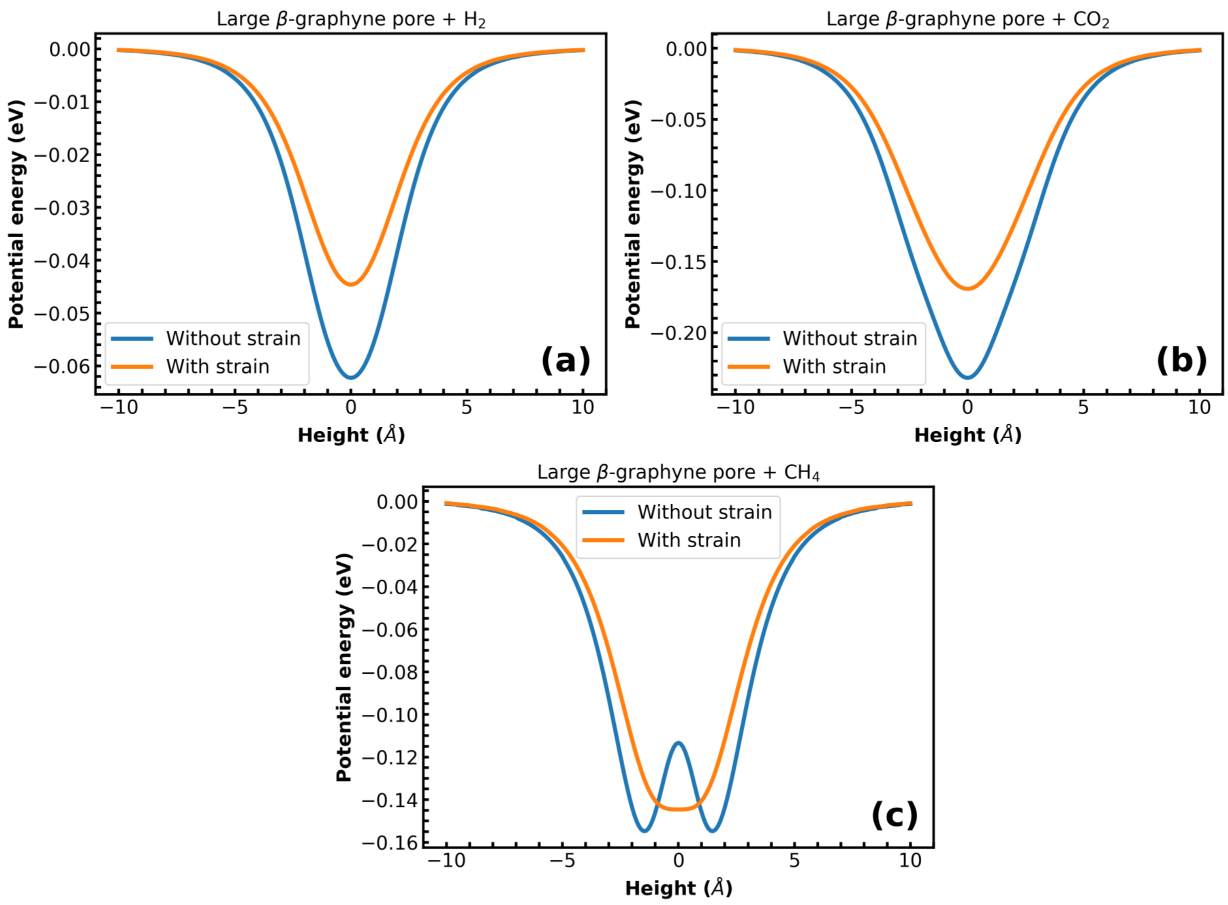

FIG. S21. Potential energy curves obtained by displacing a gas molecule through the center of the large pore of β-graphyne. For CO_2 and H_2, the molecule was aligned perpendicular to the plane of the monolayer. Since this pore is very similar to that of α-graphyne, the energy profiles are also alike. The gas-surface interactions were described using UFF for the monolayers and TraPPE for the gas molecules.

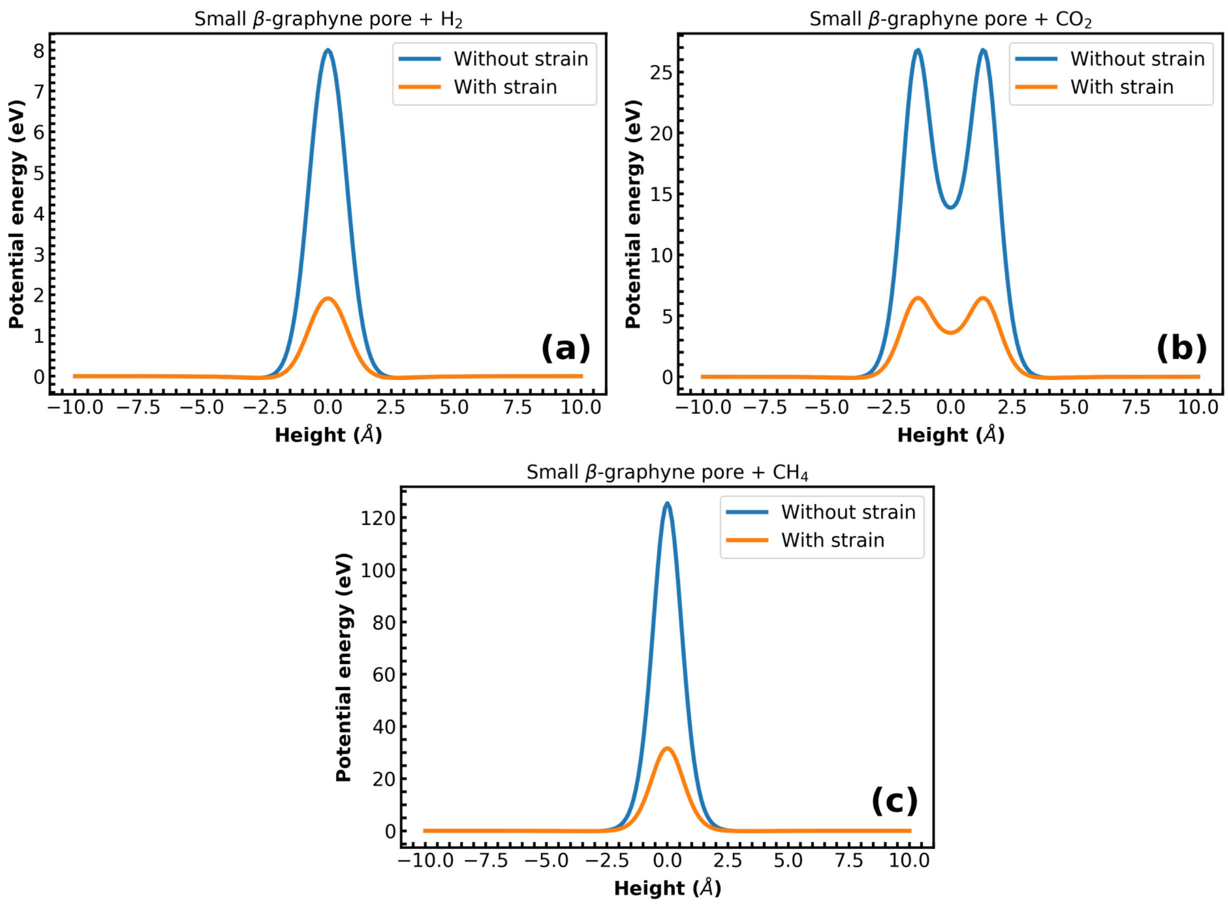

FIG. S22. Potential energy curves obtained by displacing a gas molecule through the center of the small pore of β-graphyne. For CO_2 and H_2, the molecule was aligned perpendicular to the plane of the monolayer. Since this pore is very similar to that of γ-graphyne, the energy profiles are also alike. The gas-surface interactions were described using UFF for the monolayers and TraPPE for the gas molecules.

POTENTIAL ENERGY RESULTS: COMPARING DFT AND UFF RESULTS

We performed Density Functional Theory calculations for select gas/solid combinations using the SIESTA package [5] to verify the energy profiles obtained using the Universal Force Field [6] in the previous section. In these calculations, we used a double-ζ polarized (DZP) basis set and an exchange-correlation energy functional that included van der Waals corrections [7]. In the direction perpendicular to the monolayer plane, we separated unit cells using a vacuum region of 30 Å. In all calculations, we used a mesh cutoff of 150 Ry. We assumed that self-consistency was achieved when the difference between the input and output of each element in the density matrix was smaller than 10^{-4} in a self-consistent cycle.

In the first step of these DFT calculations, we optimized a small unit cell of the considered monolayer (16 atoms for α-graphyne and 24 atoms for γ-graphyne). Using the conjugate gradient method, we optimized unit cells with and without strain, with a max force tolerance of 0.10 eV/Å and an energy tolerance of 10^{-8} Ry. For structures without strain, we also allowed the cell vectors to change. We sampled the Brillouin Zone using an $8 \times 8 \times 1$ k-point mesh using the Monkhorst and Pack scheme.

After completing the geometry optimization, for α-graphyne, we replicated the unit cell twice in the x-direction and three times in the y-direction. For γ-graphyne, we replicated the unit cell three times in the x-direction and twice in the y-direction. In both cases, the goal was to prevent interactions between periodic images of the gas molecules. We then placed a CH_4 molecule above α-graphyne and an H_2 molecule above γ-graphyne at various heights and performed single-point calculations. We considered structures with and without strain.

In the case of α-graphyne+CH_4, we wanted to verify whether there was a potential energy barrier near the monolayer plane and whether this barrier vanished as we applied strain. In the case of γ-graphyne+H_2, we wanted to ensure that the gas could not pass through its pores even after introducing deformation. The figure below presents the results.

In Fig. S23, we notice that the deformation removes the energy barrier and turns the center of the hole more attractive for CH_4 accommodation using both methods. We also find that, for heights above 2 Å, strain reduces the attractive interaction between α-graphyne and CH_4. However, deformation increases the attractive interaction near the 2D material in the DFT calculations. Furthermore, comparing the magnitude of the energy values, we see

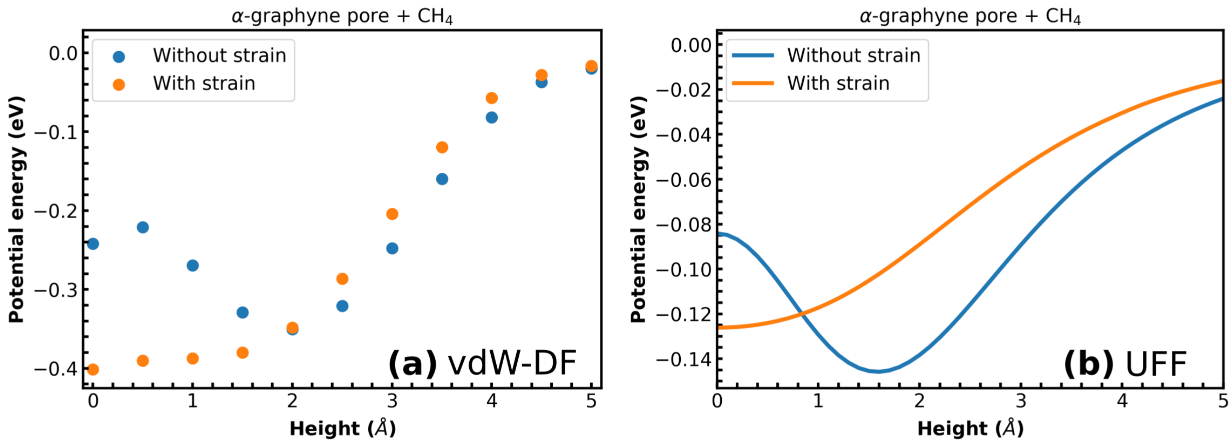

FIG. S23. Energy profile for a CH_4 molecule positioned at different heights above the center of the pore of α-graphyne. The figures in (a) and (b) display results obtained using vdW-DF and UFF, respectively.

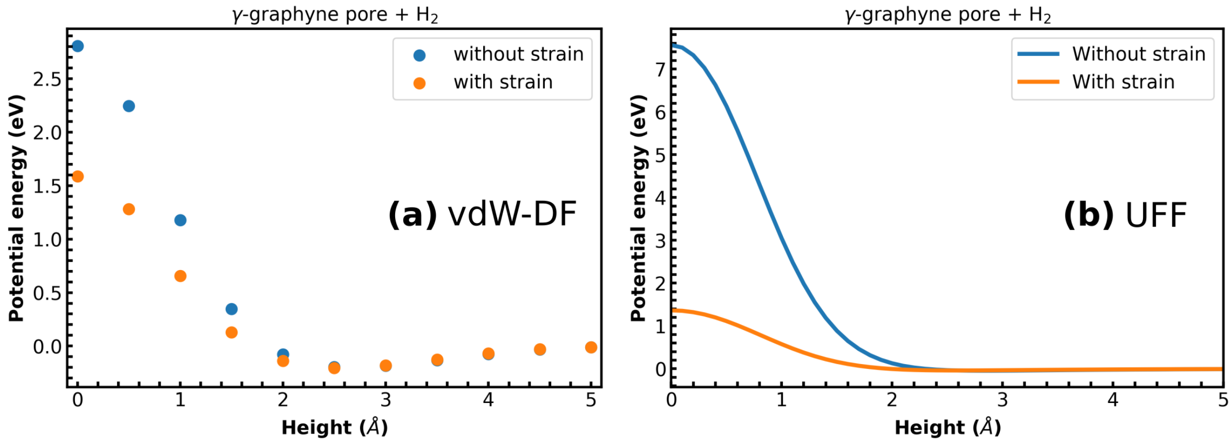

FIG. S24. Energy profile for an H_2 molecule positioned at different heights above the center of the pore of γ-graphyne. The figures in (a) and (b) display results obtained using vdW-DF and UFF, respectively.

that UFF strongly underestimates the interaction between α-graphyne and CH_4.

In Fig. S24, we find in both methods that the triangular pores of γ-graphyne are too narrow to allow gases to pass through, even after deformation. We also observe that strain reduced the magnitude of the energy barrier at the monolayer plane. However, we notice that UFF overestimates the energy barrier to pass an H_2 molecule through the triangular pores of γ-graphyne. Furthermore, the classical potential also overestimates the effect of

strain on the energy barrier.

POTENTIAL ENERGY MAPS FOR H_2 AND CO_2 NEAR THE SURFACE OF α-GRAPHYNE

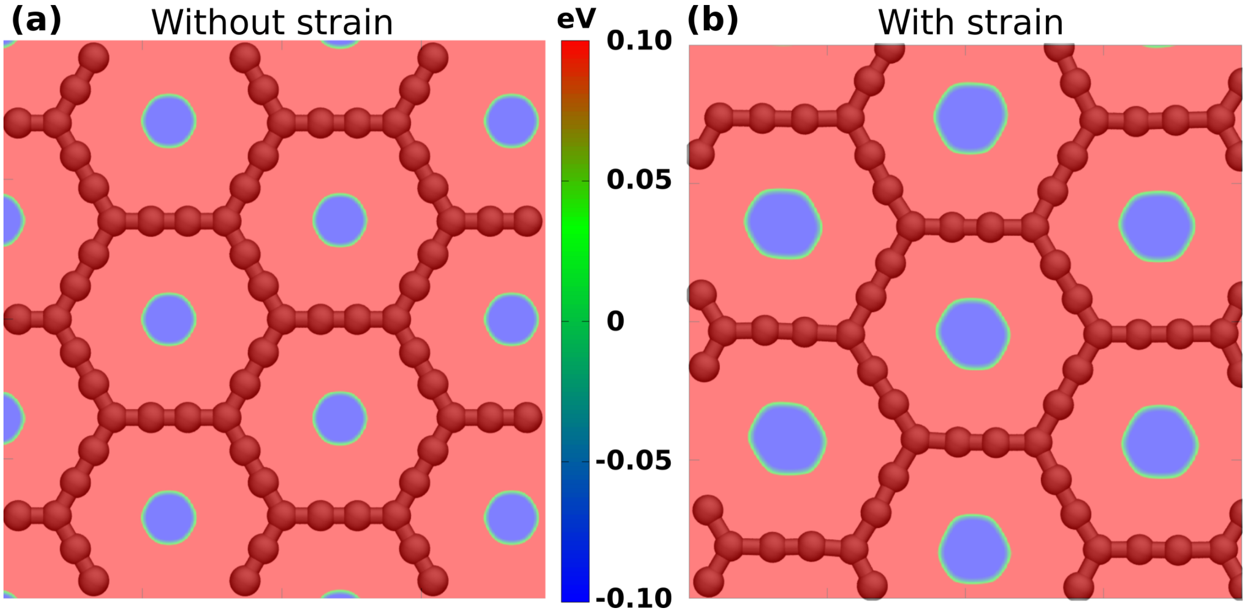

FIG. S25. Potential energy map for CO_2 above α-graphyne without (a) and with strain (b). The molecule is at a fixed height above the surface ($h = 0.5$ Å).

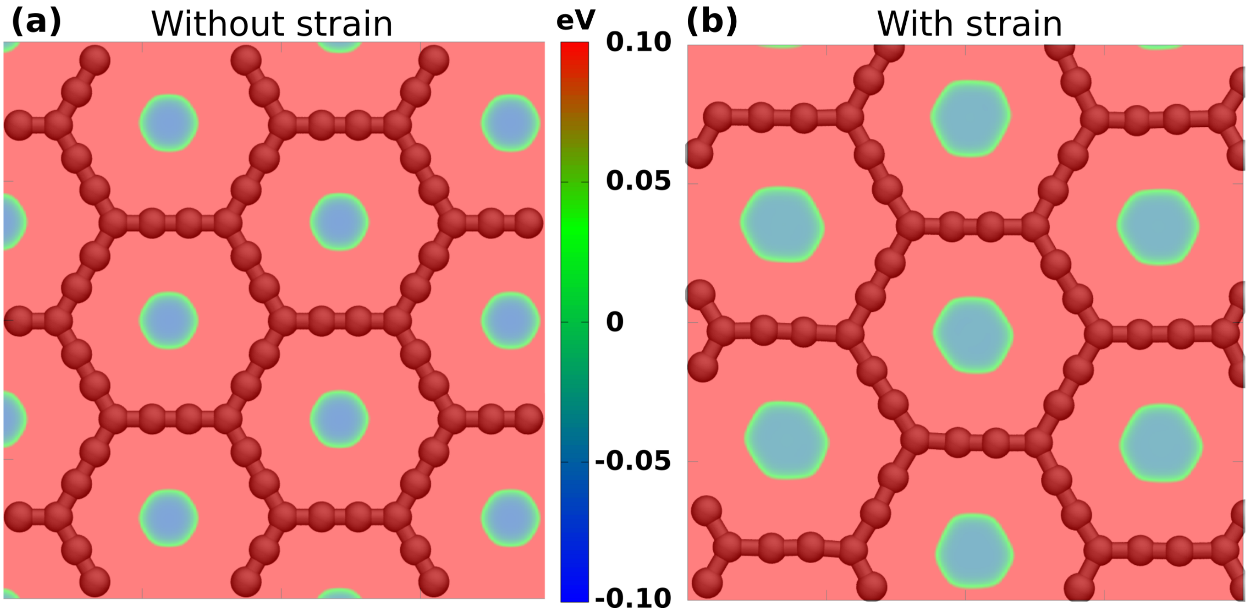

FIG. S26. Potential energy map for H_2 above α-graphyne without (a) and with strain (b). The molecule is at a fixed height above the surface ($h = 0.5$ Å).

CONSIDERING THE EFFECT OF DOMAIN SIZE ON THE ADSORPTION PROPERTIES: CHANGING THE SIZE OF THE MONOLAYER UNIT CELL

To verify whether the size of the periodic unit cell we used could influence the results, we obtained adsorption isotherms considering cells with a different number of atoms. We performed this test using CO_2 for the four monolayers, with and without strain.

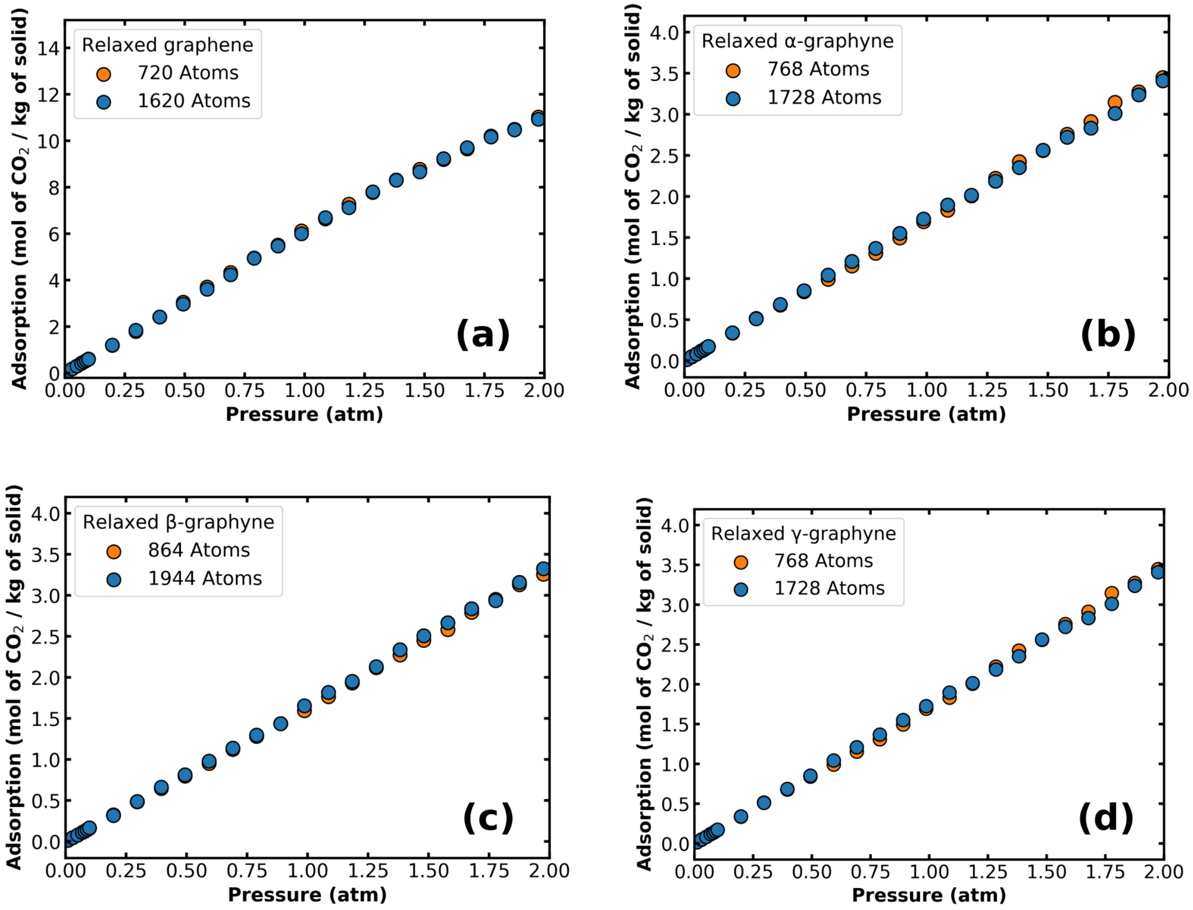

FIG. S27. In this figure, we display the results for relaxed monolayers of (a) graphene, (b) $\alpha-$graphyne, (c) $\beta-$graphyne, and (d) $\gamma-$graphyne. The data obtained suggest no apparent change in the adsorption results with unit cell size.

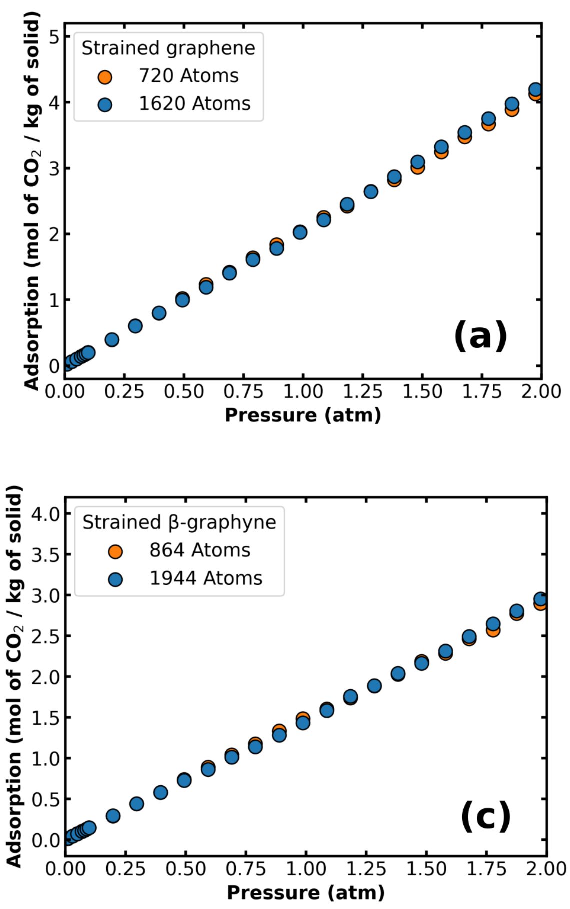

FIG. S28. In this figure, we display the results for strained monolayers of (a) graphene, (b) $\alpha-$graphyne, (c) $\beta-$graphyne, and (d) $\gamma-$graphyne. The data obtained suggest no apparent change in the adsorption results with unit cell size.

TESTING CONVERGENCE CONSIDERING DIFFERENT PROBABILITIES FOR THE MONTE CARLO MOVES.

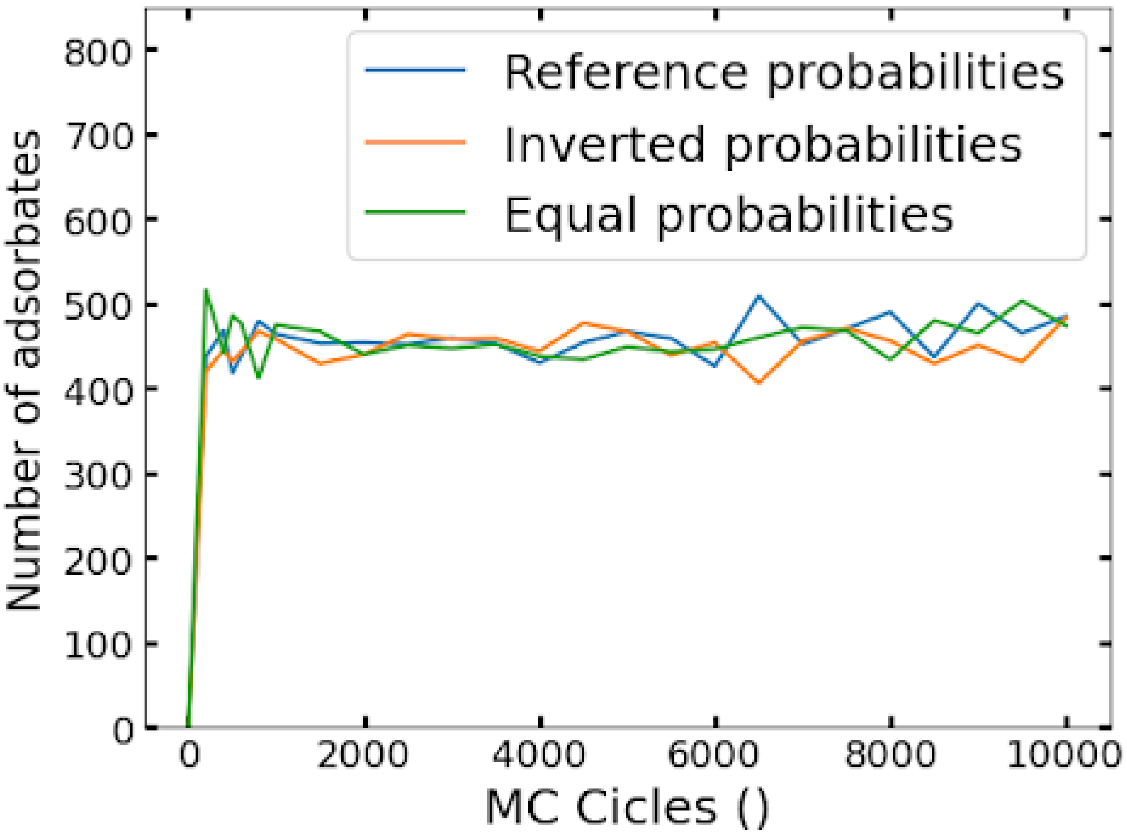

FIG. S29. Figure investigating the convergence of the number of adsorbates with the numbers of Monte Carlo (MC) cycles, using different probabilities for the MC moves. We considered the adsorption of CO_2 molecules in graphene at 50 atm and 298 K. The reference probabilities refer to those provided in the main text. In the second case, we inverted the swap probability with the rotation and translation probabilities. In the third case, we set equal probabilities for the rotation, translation, and swap operations. Convergence occurred quickly during the initialization cycles, regardless of the parameter set choice.

* leonardo@fisica.ufrn.br

[1] Q. Peng, W. Ji, and S. De, Phys. Chem. Chem. Phys. **14**, 13385 (2012).

[2] M. Raad and H. Behnejad, Journal of the Iranian Chemical Society **12**, 1999 (2015).

[3] J. Vekeman, J. Sanchez-Marin, A. Sanchez de Meras, I. Garcia Cuesta, and N. Faginas-Lago, The Journal of Physical Chemistry C **123**, 28035 (2019).

[4] C. Wang, Y. Fang, H. Duan, G. Liang, W. Li, D. Chen, and M. Long, Solid State Communications **337**, 114436 (2021).

[5] J. M. Soler, E. Artacho, J. D. Gale, A. García, J. Junquera, P. Ordejón, and D. Sánchez-Portal, Journal of Physics: Condensed Matter **14**, 2745 (2002).

[6] A. K. Rappé, C. J. Casewit, K. Colwell, W. A. Goddard III, and W. M. Skiff, J. Am. Chem. Soc. **114**, 10024 (1992).

[7] M. Dion, H. Rydberg, E. Schröder, D. C. Langreth, and B. I. Lundqvist, Physical review letters **92**, 246401 (2004).
